# Integrated analysis of microRNA and mRNA expression profiles highlights the complex and dynamic behavior of toosendanin-induced liver injury in mice

**DOI:** 10.1038/srep34225

**Published:** 2016-10-05

**Authors:** Xiaoyan Lu, Cai Ji, Wei Tong, Xueping Lian, Ying Wu, Xiaohui Fan, Yue Gao

**Affiliations:** 1 Pharmaceutical Informatics Institute, College of Pharmaceutical Sciences, Zhejiang University, Hangzhou 310058, China; 2 Department of Pharmacology and Toxicology, Beijing Institute of Radiation Medicine, Beijing 100850, China

## Abstract

Triterpenoid Toosendanin (TSN) exhibits a plenty of pharmacological effects in human and great values in agriculture. However, the hepatotoxicity caused by TSN or Melia-family plants containing TSN used in traditional Chinese medicine has been reported, and the mechanisms of TSN-induced liver injury (TILI) still remain largely unknown. In this study, the dose- and time-dependent effects of TSN on mice liver were investigated by an integrated microRNA-mRNA approach as well as the general toxicological assessments. As the results, the dose- and time-dependent liver injury and alterations in global microRNA and mRNA expressions were detected. Particularly, 9-days 80 mg/kg TSN exposure caused most serious liver injury in mice, and the hepatic adaptation to TILI was unexpectedly observed after 21-days 80 mg/kg TSN administration. Based on the pathway analysis of the intersections between predicted targets of differentially expressed microRNAs and differentially expressed mRNAs at three time points, it revealed that TILI may be caused by glutathione depletion, mitochondrial dysfunction and lipid dysmetabolism, ultimately leading to hepatocytes necrosis in liver, while liver regeneration may play an important role in the hepatic adaptation to TILI. Our results demonstrated that the integrated microRNA−mRNA approach could provide new insight into the complex and dynamic behavior of TILI.

As a kind of drug-induced liver injury (DILI), there is growing evidence suggesting that herb-induced liver injury (HILI) is an emerging cause of liver disease^1^. People usually regard herbal medicine is much safer than chemical drugs and with less side effects, but HILI is greatly increased in recently years and accounts for about 9% cases of DILI in the United States and approximately 19% to 63% cases of DILI in Asian countries^2^,^3^,^4^,^5^. Therefore, discovering the mechanism and biomarkers of HILI is of paramount importance for the safe use of herb medicines and supplements.

Toosendanin (TSN) is a main triterpenoid derivative extracted from the bark and fruit of *Melia toosendan* Sieb et Zucc that has been used as a digestive tract parasiticide and insecticide in traditional Chinese medicine^6^. Aside from traditional biological effects such as the inhibition of insect development and antibotulismic role *in vitro* and *in vivo*^6^, recent studies clearly demonstrate that TSN has been found to suppress the proliferation of mouse hepatocellular carcinoma and a variety of human cancer cells as well as hepatitis C virus replication^7^,^8^,^9^,^10^,^11^,^12^. Although TSN has the potential to use in the therapy of several liver diseases mentioned above, the TSN and Melia-family plants containing TSN used in traditional Chinese medicine have been reported to induce liver injury^13^,^14^. Previous study showed that TSN could induce cytotoxicity in primary rat hepatocytes, and the mechanism was referred to the generation of reactive oxygen species, decrease of mitochondrial membrane potential, fall in intracellular ATP level, release of cytochrome c to cytoplasm, and activation of the caspase cascade^14^. However, the precise and comprehensive toxicity mechanisms of TILI *in vivo* remain poorly clarified.

MicroRNAs (miRNAs) have recently received scientific attention for their ability to discover the mechanisms and new biomarkers of DILI. miRNAs are a class of highly conserved and short non-coding endogenous RNAs, which are 19–25 nucleotides in length. They negatively regulate gene expression by targeting complementary sequences in the 3′ untranslated region of specific mRNAs to promote degradation and/or block translation^15^. There are more than 1900 miRNAs in humans which regulate about 60% of all human genes^16^. It has been demonstrated that miRNAs are abundant in the liver and exhibit a broad range of biological functions, including proliferation and apoptosis, differentiation and development, tissue remodeling, immunity, metabolism, and intracellular signaling^17^. The importance of miRNAs in DILI is also being increasingly recognized. For instance, miR-122 and miR-192 in circulation have demonstrated as promising biomarkers for the prediction of DILI in preclinical species and in patients^18^.

Moreover, the integrated miRNA−mRNA approach has been demonstrated as a valuable platform for identifying molecular mechanisms of DILI, especially focusing on the predicted targets of differentially expressed miRNAs (DEMs) that were also differentially expressed following exposure to drugs^19^,^20^. For example, Lizarraga *et al*.^19^ found that possible miRNA−mRNA interactions involved in relevant genotoxic response pathways provided new insight into the action mechanism of benzo[α]pyrene (BaP)-induced toxicity, indicating the potential of BaP as a genotoxic carcinogens. Our previous studies also used this approach to reveal the mechanisms of hepatotoxicity caused by water and ethyl acetate extracts of Fructus Meliae Toosendan (FMT)^21^,^22^ which is a traditional Chinese medicine containing TSN as an active component.

Thus, the objective of this study is to clarify the mode of action that operates in liver following TSN exposure via oral gavage. The dose- and time-dependent effects of TSN on mice liver were determined by two experiments. One was set to address the dose-dependent effects of TSN in liver, i.e., 40 and 80 mg/kg TSN treatments for 9 days (experiment 1, n = 8). The second was to investigate the time-dependent toxicity of TSN, i.e., 80 mg/kg TSN treatment for 3, 9 or 21 days (experiment 2, n = 8). miRNA and mRNA expression profiles were both investigated in TSN-treated mice as well as the general toxicological assessments. The integrated miRNA−mRNA approach was applied to provide new insight into the mechanism of TILI. Unexpectedly, the hepatic adaptation to TSN toxicity was observed in the time-dependent exposure of TSN, indicating the complex and dynamic behavior of TILI.

## Results

### The dose- and time-dependent effects of TSN on mouse body weight and serum parameters

**Figure 1:**
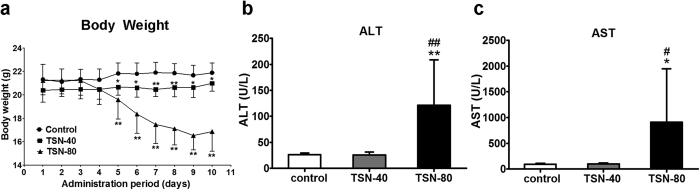
The effects of 40 and 80 mg/kg TSN on the general toxicological parameters. (**a**) The body weight of the mice exposed to 40 and 80 mg/kg TSN for 9 days. Individual body weight was recorded every day until day 10. (**b,c**) The serum ALT (**b**) and AST (**c**) activities of the mice exposed to 40 and 80 mg/kg TSN for 9 days. TSN-40 indicates 40 mg/kg TSN treatment, and TSN-80 represents 80 mg/kg TSN exposure. *p < 0.05, **p < 0.01 compared with control group; ^#^*p* < 0.05, ^##^*p* < 0.01 compared with 40 mg/kg TSN treatment.

In experiment 1, the body weight was significantly affected by high dose TSN treatment. As shown in Fig. 1a, a marked decrease was detected after 80 mg/kg TSN exposure from day 5 to day 10 compared with vehicle control group. The statistical significance was also observed between 40 mg/kg TSN-treated and vehicle control groups from day 5 to day 10, but there was no physiological difference as the body weight curves were similar in these two groups. Furthermore, serum biochemistry was analyzed to determine the hepatic effects of TSN. The results are shown in Fig. 1b,c. Administration of 80 mg/kg TSN for nine consecutive days by oral gavage resulted in a significant increase in serum alanine transaminase (ALT) and aspartate transaminase (AST) levels, whereas no effects were detected for these two parameters after 40 mg/kg TSN treatment, indicating the liver toxicity induced by TSN was dose-dependent. The levels of alkaline phosphatase (ALP), total bilirubin (TBILI), total cholesterol (TCHOL), and triglyceride (TG) in serum did not markedly change after 40 or 80 mg/kg TSN exposure for 9 days compared with vehicle control group (Supplementary Fig. S1).

**Figure 2:**
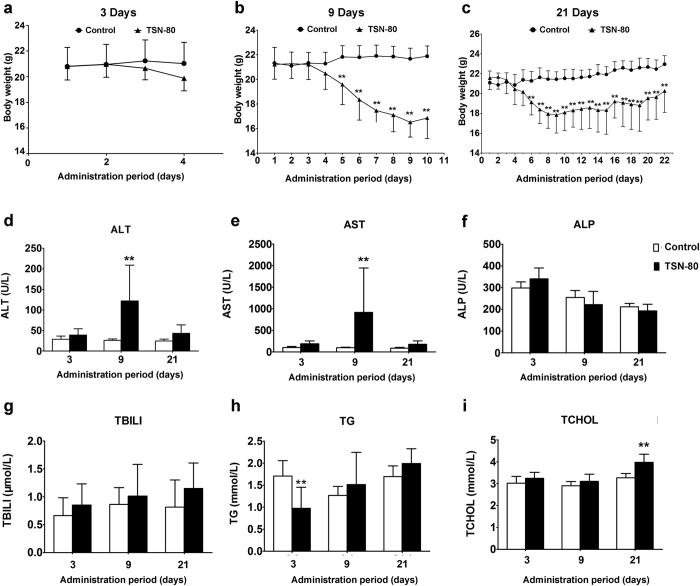
The time-dependent effects of TSN on mouse body weight and serum parameters. (**a–c**) The body weight of the mice exposed to 80 mg/kg TSN for 3 (**a**), 9 (**b**), or 21 days (**c**). Individual body weight was recorded every day until day 4 (**a**), day 10 (**b**), or day 22 (**c**). (**d–i**) The serum parameters of the mice exposed to 80 mg/kg TSN for 3, 9, or 21 days. (**d**) ALT, (**e**) AST, (**f**) ALP, (**g**) TBILI, (**h**) TG, (**i**) TCHOL. TSN-80 represents 80 mg/kg TSN exposure. **p < 0.01 compared with control group.

In experiment 2, a slight decrease of the body weight was noted after 3-days 80 mg/kg TSN administration, while a marked decrease was detected in 9-days exposure from day 5 to day 10 as mentioned above (Fig. 2a,b). Most interesting was shown in 21-days treatment as the body weight was unexpectedly increased after 9-days administration, although the mice were still exposed to TSN (Fig. 2c). In serum, significant increases were only observed in 9-days treatment for ALT and AST activities, with no obvious change detected by 3-days and 21-days TSN treatments on these two biomarkers of liver injury. Similarly to experiment 1, there were no significant effects identified for ALP, TBILI, TG, and TCHOL levels in experiment 2, except the decrease in TG level after 3-days TSN treatment and the marked increase in TCHOL concentration after 21-days TSN exposure.

### The dose- and time-dependent effects of TSN on liver histopathology

**Figure 3:**
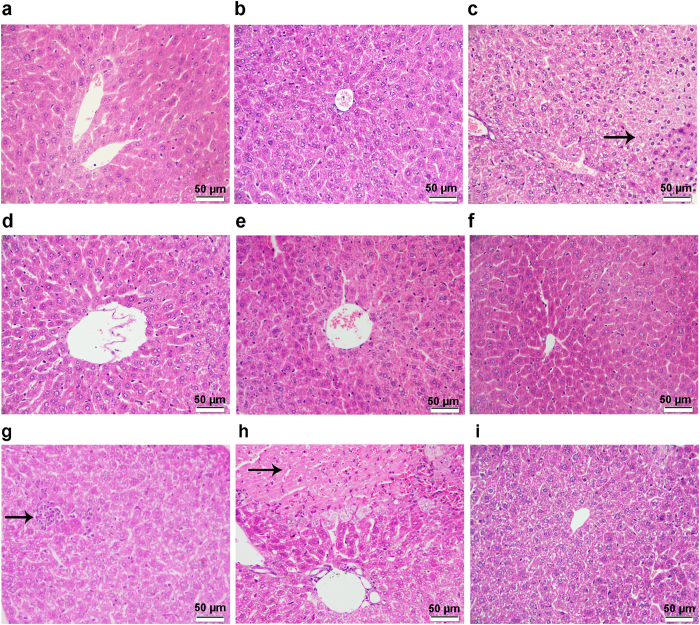
Photomicrographs of liver tissues of the mice in experiments 1 and 2 with hematoxylin and eosin staining. (**a–c**) Liver tissues of the mice in experiment 1. (**a**) Control, (**b**) 9-days 40 mg/kg TSN exposure,(**c**) 9-days 80 mg/kg TSN treatment. (**d–i**) Liver tissues of the mice in experiment 2. (**d–f**) Liver tissues of the mice from control groups: (**d**) 3 days, (**e**) 9 days, (**f**) 21 days; liver tissues of the mice from 80 mg/kg TSN-treated groups: (**g**) 3 days, (**h**) 9 days, (**i**) 21 days. The magnification used was 400×. The scale bar is 50 μm. The arrow in (**g**) showed the inflammation in the livers, and the arrow in (**c**,**h**) indicated the necrotic hepatocytes.

In order to verify the results of serum parameters, the histopathological examinations were performed to TSN treatments as well as the vehicle controls. In experiment 1, administration of 80 mg/kg TSN for 9 days caused hepatocytes necrosis, whereas the livers of vehicle control group exhibited normal histology and the livers of 40 mg/kg TSN treatment displayed slight hydropic degeneration of hepatocytes after 9 days exposure (Fig. 3a–c). This was consistent with the dose-dependent manner in body weight and serum ALT and AST activities after 40 and 80 mg/kg TSN treatments.

In experiment 2, a time-dependent response on liver histopathology was observed after 80 mg/kg TSN exposures (Fig. 3d–i). Mice treated with TSN for 9 days revealed most serious liver injury as the hepatocytes necrosis was detected, while the vehicle control at all time points showed normal morphology and 3-days TSN exposure displayed mild hydropic degeneration of hepatocytes and inflammatory foci in livers. Interestingly, 21-days TSN administration did not cause most serious liver injury, and only slight hydropic degeneration of hepatocytes was noted, which was consistent with the recovery trend in body weight and serum ALT and AST levels.

### The dose-dependent effects on mRNA and miRNA expression profiles

In experiment 1, exposure to TSN caused a large response in mRNA and miRNA expressions in liver. Particularly, 50 and 807 mRNAs were differentially expressed in the 40 and 80 mg/kg exposures, respectively (Supplementary Table S1). Twelve differentially expressed mRNAs (DEGs) were overlapped between these two dosage treatments (Supplementary Table S2). Among them, *Fmo3* showed the greatest fold-change in expression after TSN treatments, with upregulated over 5-fold after 40 mg/kg TSN treatment and 268-fold after 80 mg/kg TSN exposure. This gene encoding a protein belongs to flavin-containing monooxygenases (FMO) family which are an important class of drug-metabolizing enzymes that catalyze the nicotinamide adenine dinucleotide phosphate (NADPH)-dependent oxygenation of various nitrogen-, sulfur-, and phosphorous-containing xenobiotics. Moreover, significantly increased levels of several metabolism-related genes was also observed in these common DEGs, including *Acox1* (peroxisomal acyl-coenzyme A oxidase 1), *Srebf1* (sterol regulatory element binding transcription factor 1), *Acnat2* (acyl-coenzyme A amino acid N-acyltransferase 2), and *Hmgcr* (3-hydroxy-3-methylglutaryl-coenzyme A reductase).

In addition, 14 miRNAs in 80 mg/kg and 25 miRNAs in 40 mg/kg TSN groups were significantly regulated (*p* value < 0.05 and absolute fold change > 1.5, Supplementary Table S1). Among them, 4 miRNAs were induced in common between these two dosage exposures, i.e., miR-504-3p, miR-1927, miR-6401, and miR-1892.

**Figure 4:**
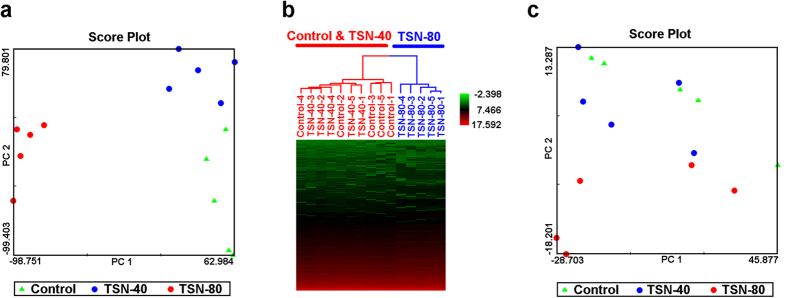
The dose-dependent effects of TSN on the mRNA and miRNA expression profiles. (**a,b**) The PCA (**a**) and HCA (**b**) score plots of the liver samples from the mice exposed to 40 and 80 mg/kg TSN for 9 days based on the global mRNA expression profiles. (**c**) The PCA score plot of the liver samples from the mice exposed to 40 and 80 mg/kg TSN for 9 days based on the global miRNA expression profiles.

In order to explore expression pattern and dose-response relationship, principal component analysis (PCA) and hierarchical clustering analysis (HCA) were used to visualize the clusters of samples corresponding to the global mRNA or miRNA gene expression profiles in experiment 1. For mRNA expression profiles, a 2-dimensional (2D) PCA view is displayed in Fig. 4a, using the first two principal components for the global gene expression data from the samples of vehicle control, 40, and 80 mg/kg TSN-treated groups. The plot showed that all of the control and 40 mg/kg TSN-treated samples were clustered on the right, whereas all the samples of 80 mg/kg TSN exposure were clearly separated from this cloud and located on the left. A counterclockwise trend was detected for the clusters of samples from vehicle control, 40, and 80 mg kg TSN exposures, indicating a dose-dependent response in mRNA expression after TSN treatments. The clustering result of HCA was corresponding to PCA (Fig. 4b). For miRNA expression profiles, although separation was less pronounced than mRNA expression profiles in PCA score plot, the dose-dependent manner was also noted for these three treatments (Fig. 4c).

### The time-dependent effects on mRNA and miRNA expression profiles

**Figure 5:**
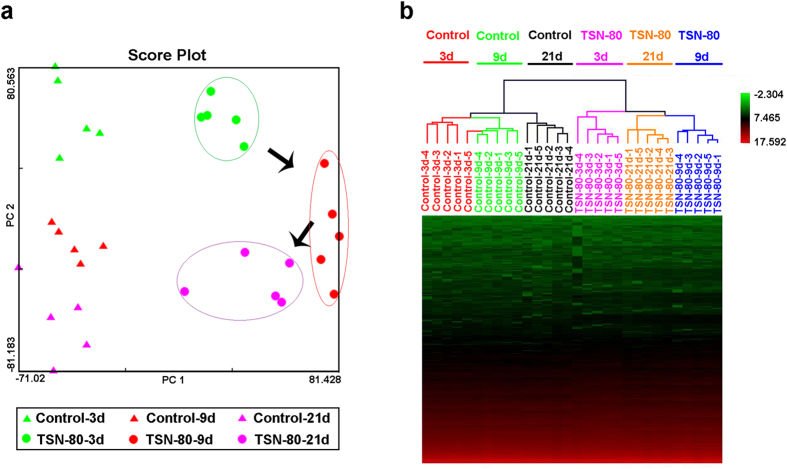
The time-dependent effects of TSN on the mRNA expression profiles. (**a,b**) The PCA (**a**) and HCA (**b**) score plots of the liver samples from the mice exposed to 80 mg/kg TSN for 3, 9 or 21 days based on the data of global mRNA expression profiles.

In experiment 2, a time-dependent response on mRNA and miRNA expression profiles in response to TSN was observed. In particular, 266, 807, and 565 mRNAs were differentially expressed in the 3-, 9-, and 21-days 80 mg/kg TSN exposures, respectively (Supplementary Table S1). There were 57 DEGs induced in common among these three time point treatments. The detail changes of these genes were shown in Supplementary Table S3. The majority of these DEGs had a time-dependent expression trend as the most significant change was detected after 9-days treatment, while *Fmo3* was the gene of greatest significant change after 9- and 21-days exposures, with upregulated 268.2-fold after 9-days 80 mg/kg TSN treatments and 215.0-fold after 21-days administration. The time-dependent effects were also detected in the PCA and HCA results. The score plot of PCA showed that all the samples from control groups of three time points were gathered in the left, while the samples of 80 mg/kg TSN-treated groups at three time points were separated from this cloud and moved to the right (Fig. 5a). Among the three TSN-treated groups, it is worth noting that the samples of 9-days TSN exposure reached maximum shift from control cloud, whereas the samples of 3 and 21 days TSN exposures in the middle of these two clusters (Fig. 5a). Similarly, the clustering result of HCA had the same trend (Fig. 5b), which was consistent with general toxicological data mentioned above as 9-days TSN treatment induced most serious liver injury among the three time point exposures in experiment 2.

Moreover, there were 43, 14, and 69 differentially expressed miRNAs (DEMs, *p* value < 0.05 and absolute fold change > 1.5) observed after 3-, 9-, and 21-days 80 mg/kg TSN treatments, respectively (Supplementary Table S1). However, no common DEMs were detected among the three time points, indicating the complex and dynamic behavior in miRNAs expression to TILI. Only 17 miRNAs were differed significantly in the same manner between either two time points (Supplementary Table S4). More interestedly, there were more DEMs (14 DEMs) induced in common between 3- and 21-days exposures, suggesting similar pathological and physiological processes were occurred in these two treatments. As shown in Supplementary Fig. S2, the score plot of PCA showed a clear separation between control and TSN-treated groups at any time point for the miRNAs expression profiles in experiment 2.

### Verification of TSN-induced changes in gene expression by real time quantitative reverse transcriptase polymerase chain reaction analysis (qRT-PCR)

**Figure 6:**
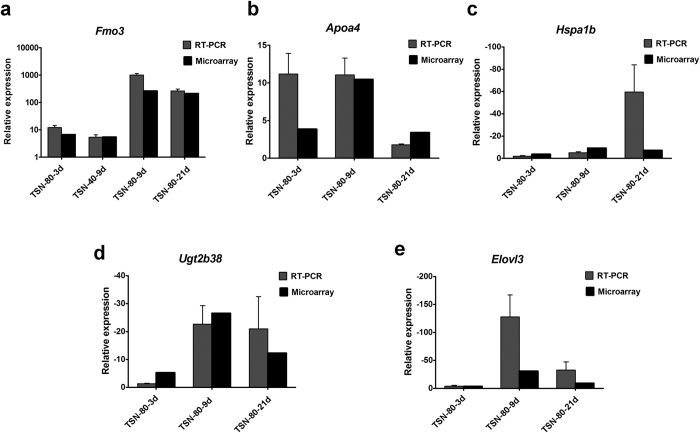
Validation of the microarray results by quantitative RT-PCR. (**a**) *Fmo3*, (**b**) *Apoa4*, (**c**) *Hspa1b*, (**d**) *Ugt2b38*, (**e**) *Elovl3*. Black bars indicate microarray data (if the probes with same genename/LocusID were identified, the largest fold change was indicated in this figure). Gray bars represent mean fold change (±SD) derived from 3 independent experiments performed in duplicate of qRT-PCR experiments.

The general trends of dose- or time-dependent response observed in the microarray analysis of all the genes tested were verified by qRT-PCR (Fig. 6). The qRT-PCR analysis showed that *Fmo3*, the drug-metabolizing related gene, was upregulated in 40 and 80 mg/kg of TSN treatments at all tested time points, and the expression levels of *Apoa4* were also increased after 3-, 9- and 21-days TSN exposures at 80 mg/kg. Moreover, the fold change values decreased in the qRT-PCR of the genes including *Hspa1b*, *Ugtab38*, and *Elovl3*, which showed similar patterns with microarray data.

Furthermore, the expression levels of 8 DEMs (i.e., miRNA-1927, miRNA-802-5p, miRNA-6236, miRNA-3968, miRNA-126-5p, miRNA-100-5p, miRNA-5100, and miRNA-3102-3p) were also verified by qRT-PCR (Supplementary Table S5). The only exception was observed on the levels of miR-3968 after 21-days 80 mg/kg TSN treatment, which showed an opposite trend of expression between microarray analysis and qRT-PCR.

### Functional analyses of the miRNA-mRNA intersection dataset

We used IPA to predict targets of the DEMs in 80 mg/kg TSN-treated group at each time point and also generated miRNA-mRNA intersection dataset based on the approach mentioned in “Materials and Methods”. As shown in Table 1, for 3-days treatment, 16 DEMs had targets information in IPA. Among these predicted targets, 25 genes were the DEGs from mRNA profile and expressed in the opposite direction to their respective miRNAs following TSN treatment at 3 days. Thus, the miRNA-mRNA intersection dataset for 3-days TSN treatment was the 25 DEGs. In the same way, 4 DEMs were related to 45 target-DEGs which served as the miRNA-mRNA intersection dataset for 9-days exposure, while 32 miRNAs were corresponding to 190 target-DEGs after 21-days TSN treatment (Table 1). This analysis relies on the speculation that the predicted targets that are changing in the opposite direction of their miRNAs are likely controlled by these miRNAs. The detail information of these miRNA-mRNA intersections was provided in Supplementary Tables S6–8, including name, fold change, *p* value, and confidence of target mRNA’s prediction.

Moreover, the “tox analysis” function in IPA was performed to analysis the pathways of these targets from each time point. The top five pathways in “Molecular and Cellular Functions” affected by 80 mg/kg TSN treatments of three time points were shown in Supplementary Table S9. For 3-days treatment, these direct miRNA targets were mainly related to the pathways of cellular development, growth and proliferation, function and maintenance, cell death and survival, and cell cycle; for 9- and 21-days exposures, the direct miRNA targets were primarily associated with lipid metabolism, small molecule biochemistry, and molecular transport, whereas energy production and cell-to-cell signaling and interaction were the specific pathways for 9 days, and cellular growth and proliferation and cell death and survival were also significantly affected for 21-days TSN treatment.

**Figure 7:**
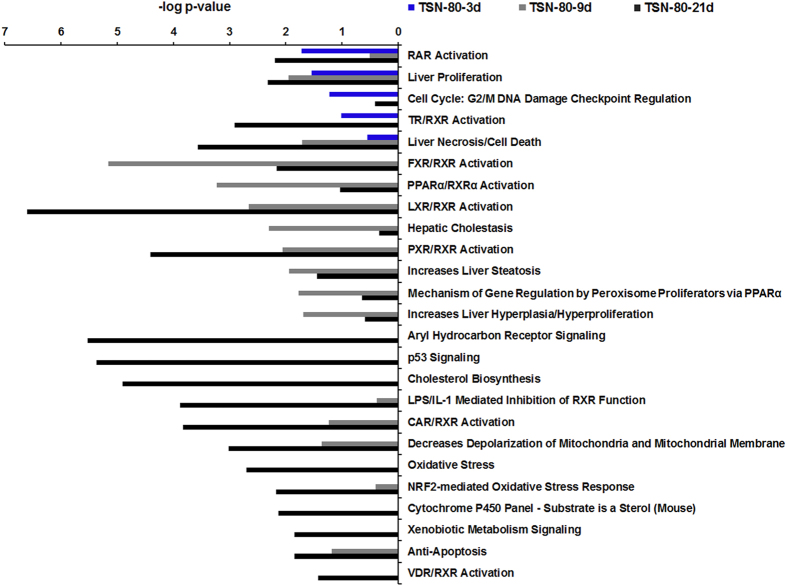
The 25 characteristic pathways associated with TILI were identified by the target mRNAs of miRNA-mRNA intersection after the time-course exposures of 80 mg/kg TSN.

In addition, based on the −log *p* value in “Tox lists” by the “tox analysis” in IPA, the target mRNAs related pathways at each time point were sorted in descending order. The significantly enriched pathways were detected based on −log *p* value greater than 1.30 (i.e., *p* < 0.05) in the three time points. However, some of these significant pathways were related to cardiac and renal diseases, such as cardiac hypertrophy, cardiac fibrosis, and acute renal failure panel. Thus, these pathways were not considered as the characteristic pathways of TILI. By removing the duplicates and cardiac and renal diseases related pathways, 25 characteristic pathways were identified after TSN exposures in the three time points. The −log *p* values of these 25 pathways for the three time points were all given in Fig. 7 even though the pathways in the specific time point were not significantly enriched. As shown in Fig. 7, RAR (retinoic acid receptor) activation, liver proliferation, and liver necrosis/cell death were all affected by three time points, whereas aryl hydrocarbon receptor (AhR) signaling, p53 signaling, cholesterol biosynthesis, oxidative stress, cytochrome P450 panel-substrate is a sterol, xenobiotic metabolism signaling, and VDR/RXR (vitamin D receptor/retinoid X receptor) activation were only affected by 21-days TSN treatments, and the pathways of FXR (farnesoid X receptor)/RXR activation, peroxisome proliferator-activated receptors alpha (PPARα)/RXRα activation, hepatic cholestasis, increases liver steatosis, mechanism of gene regulation by peroxisome proliferators via PPARα, and increases liver hyperplasia/hyperproliferation were identified by the most large −log *p* values by 9-days TSN administration.

## Discussion

This study is the first time to report the dose- and time-specific alterations in global miRNA and mRNA expressions after exposed to TSN. The liver injury induced by TSN was confirmed from the results of general toxicological assessments. Moreover, the complex and dynamic behavior of TILI in mice was revealed by microRNA and mRNA expression profiles as well as the traditional toxicological parameters. In particular, exposure to 80 mg/kg TSN for 9 days caused most serious liver injury in mice, and a recovery trend was detected after 21-days TSN treatment.

It was reported that TSN contains a furan ring which serves as a structural alert^23^. The toxicity of several furan-containing compounds was attributed to a bioactivated furan ring, such as teucrin A, 4-ipomeanol, and aflatoxin B1^24^,^25^,^26^,^27^. Previous study reported that bioactivation of the furan ring of TSN was mediated by CYP3A4 and yielded a *cis*-butene-1,4-dial intermediate^28^. The latter might be responsible for TILI, while glutathione (GSH) plays an important role in the elimination of reactive metabolites of TSN^28^. Consistently, in this study, the pathway of GSH Depletion was significantly induced by 9-days 80 mg/kg TSN exposure in mRNA expression profiles due to the genes of *Gsta5*, *Gstm6*, and *Xdh* (detail data not shown), indicating GSH depletion may be the first step of TILI. Meanwhile, the NRF2-mediated oxidative stress response which is involved in the antioxidation reaction^29^ was affected by 9-days 80 mg/kg TSN exposure (*p* > 0.05) and significantly induced by 21-days treatment (*p* < 0.05, Fig. 7), further suggesting oxidative stress occurred after TSN administration. Moreover, previous study showed that oxidative stress caused by TSN could lead to mitochondrial dysfunction in primary rat hepatocytes^14^, and mitochondrial disturbances can have varieties of deleterious consequences, including steatosis, energy shortage, and cell death^30^. In this study, the pathway of decreases depolarization of mitochondria and mitochondrial membrane was markedly induced and hepatocytes necrosis was detected after 9-days 80 mg/kg TSN exposure (Figs 3 and 7), suggesting the mitochondrial dysfunction may be occurred. Meanwhile, energy production was also significantly induced in “Molecular and Cellular Functions” by 9-days 80 mg/kg TSN treatment (Supplementary Table S9), further indicating mitochondrial dysfunction. On the other hand, lipid dysmetabolism may also be one of the critical steps in TILI as the pathways of nuclear hormone receptors involved in regulation of hepatic lipid metabolism were significantly activated by 9-days 80 mg/kg TSN exposure, such as FXR/RXR, LXR/RXR, and PPARα/RXRα^31^ (Fig. 7). The greatest induction of the pathways including hepatic cholestasis and increases liver steatosis in 9-days 80 mg/kg TSN treatment also indicated the lipid dysmetabolism (Fig. 7). Typically, the hepatic steatosis related genes, *Apoa4*^32^ and *Elovl3*^33^, showed greater regulation by 9-days 80 mg/kg TSN exposure and had a decrease trend by 21-days treatment (Supplementary Table S3), which were verified by qRT-PCR (Fig. 6) and suggested the most serious liver injury occurred by 9-days administration. Additionally, the pathways of RAR activation, liver proliferation, and liver necrosis/cell death were all affected by three time points of 80 mg/kg TSN exposures (Fig. 7). Retinoic acid is an important regulator of cell proliferation and differentiation, which binds to two members of the nuclear receptor superfamily, RAR and RXR. Previous study showed clear evidence of *in vivo* release of RAR-α and RXR-α as insoluble lipid droplets in the vicinity of necrotic areas in chemical-induced liver injury^34^, suggesting RAR activation in this study was also related to cell proliferation and death. Thus, the TILI may be caused by GSH depletion, mitochondrial disturbances and lipid dysmetabolism, ultimately leading to hepatocytes necrosis in liver. Consistently, hepatotoxicity induced by aflatoxin B1 (furan-containing compound) also related to GSH depletion and oxidative stress with subsequent mitochondrial dysfunction and lipid metabolism disorder revealed by our previous transcriptomics and metabonomics analyses^35^, possibly suggesting the similar toxicity mechanisms of this structural alert.

More importantly, in the time-specific responses, a recovery trend on liver toxicity was detected after 21-days 80 mg/kg TSN exposure rather than expected acceleration (Figs 2, 3 and 5), indicating the hepatic autoprotection or adaptation to TILI. The same phenomenon in DILI were also reported previously by a few studies, including the drugs of APAP^36^,^37^, carbon tetrachloride^38^, and isoniazid^39^, but the knowledge on the mechanism of hepatic adaptation to DILI is very limited. It was reported that compensatory liver regeneration plays a critical role in determination of final outcome of chemical-induced liver injury and DILI^40^. For instance, previous study showed that mice treated with 300 mg/kg APAP developed extensive liver injury and robust liver regeneration^40^. Thus, we speculated that the recovery trend in 21 days may be caused by liver regeneration. Accordingly, the pathways of cellular growth and proliferation and cell death and survival were significantly affected in “Molecular and Cellular Functions” for 21-days TSN exposure (Supplementary Table S9), and cholesterol biosynthesis, p53 signaling, AhR signaling, oxidative stress, cytochrome P450 panel-substrate is a sterol, xenobiotic metabolism signaling, and VDR/RXR activation were only affected in this group in “Tox lists” (Fig. 7), indicating these pathways may be involved in the liver regeneration by sustained TSN exposure. Cholesterol is a structural component of biological membranes that modulates membrane permeability and fluidity as well as membrane proteins, protein trafficking, and transmembrane signalling^41^. Importantly, cholesterol is also a precursor for the generation of steroids and bile acids, which contribute to the solubilization of other lipids and function as signal transducers^42^. It demonstrated that alterations in hepatic and circulating pools of cholesterol may play a critical role in liver regeneration^43^,^44^. For instance, Zaid *et al*.^43^ detected that the proprotein convertase subtilisin/kexin type 9 knockout mice exhibit decreased pools of circulating cholesterol, impaired regeneration, and hepatic necrosis after partial hepatectomy (PH), but all of the animals are reversed by high cholesterol feeding; Lo Sasso *et al*.^44^ reported that cholesterol biosynthesis was stimulated during liver regeneration in the mouse model of PH. Thus, induction of cholesterol biosynthesis in 21-days TSN treatment was indicative of liver regeneration occurred. It was consistent with the marked increase in serum TCHOL concentration by 21-days TSN exposure (Fig. 2). Next, AhR signalling was also only affected by 21-days TSN treatment. The disturbed genes were referred to *Gstm1*, *Src*, *Cyp3A5*, *Fasn*, *Cdkn1a*, *Cdk4*, *Hsp90aa1*, *Rbl1*, *Ccnd1*, and *Mcm7*. Among them, *Cdkn1a* (also known as p21), *Cdk4*, and *Ccnd1* were also induced in p53 signaling, which were all increased after drug treatment. Previous study demonstrated that sustained AhR activity can induce cell cycle arrest and ultimately attenuates liver regeneration *in vivo*^45^. However, other studies showed that activation of AhR induced Fmo3 mRNA and protein levels^46^,^47^, and the induction of Fmo3 plays a positive role in the autoprotection of APAP-induced liver injury^36^. In this study, this gene was also upregulated after all TSN treatments and maintained in an extremely higher expression levels by 9- and 21-days 80 mg/kg TSN exposures (268.2-fold in 9 days and 215.0-fold in 21 days), suggesting Fmo3 might be associated with the autoprotection to TILI. Although the precise function of AhR signalling in the adaptation to TILI needs to be further investigated, this pathway might be participant in the liver regeneration caused by TSN. Moreover, it demonstrated that induction of cyclin D1 and CDK4 proteins at the early stage resulted in timely liver regeneration and recovery after 300 mg/kg APAP exposure in mice, while the mRNA and protein levels of p21 were also markedly induced at this stage^40^, which was consistent with the expression trends of these genes in this study. Another literature revealed that the increased levels of p53 and its downstream target p21 protein were associated with compensatory liver regeneration after APAP-induced acute liver injury, and p53 production may contribute to the proliferation of rat hepatocytes^48^,^49^, suggesting p53 signaling may have positive effects in the liver regeneration by sustained TSN exposure. In addition, the pathways of oxidative stress, cytochrome P450 panel-substrate is a sterol, xenobiotic metabolism signaling, and VDR/RXR activation were also only induced in 21-days treatment. The target genes in the first three pathways were mainly related to cholesterol metabolism (such as *Cyp39A1* and *Cyp51A1*) and detoxification response (such as *Gstm1* and *Abcb1*). For instance, *Gstm1* encodes a glutathione S-transferase that functions in the detoxification of electrophilic compounds, and *Abcb1* encodes a protein that is an ATP-dependent drug efflux pump for xenobiotic compounds; the upregulation of these genes in 21-days treatment indicated that detoxification response may be enhanced in this period (Supplementary Table S8); meanwhile, the increased expressions of *Cyp39A1* and *Cyp51A1* were referred to affect cholesterol metabolism as the former is involved in the conversion of cholesterol to bile acids and the latter participates in the synthesis of cholesterol (Supplementary Table S8). Furthermore, it reported that VDR has anti-fibrotic properties and VDR ligands serve as therapeutic agents in the treatment of chronic liver disease^50^, suggesting the positive role of VDR/RXR activation in the recovery trend by 21-days exposure. Taken together, the results from our study indicated these pathways and genes play a critical role in hepatic adaptation to TILI although the precise protection mechanism remains to be elucidated.

Additionally, TSN is the main toxic component of FMT^14^. Thus, in order to discover the potential biomarkers to predict the FMT-induced liver injury (FMT-ILI), we used Venn diagram to realize the overlap genes among the miRNA-mRNA intersections between this study and our previous studies of FMT water and ethyl acetate extracts-induced liver injury^21^,^22^. The miRNA-mRNA intersections of FMT water and ethyl acetate extracts-induced liver injury were based on our previous studies^21^,^22^, and the miRNA-mRNA intersection of 9-days 80 mg/kg TSN treatment in this study was used to generate the Venn diagram as this exposure caused most serious liver injury. The results showed that two genes were induced in common (Supplementary Fig. S3), i.e., *Mcl1* and *Tox*. They were all downregulated in these three treatments, indicating the two genes play important roles in FMT-ILI. *Mcl1* encodes induced myeloid leukemia cell differentiation protein (Mcl-1) which is a crucial anti-apoptotic protein in the Bcl-2 family, and Mcl-1-deficient hepatocytes are prone to undergo apoptosis^51^. Previous study showed that the up-regulation of Mcl-1 prevents Fas-induced hepatocyte apoptosis and liver injury^51^. Thus, the down-regulation of *Mcl1* in our studies may be served as a potential biomarker for FMT-ILI. Aliahmad *et al*.^52^ demonstrated that nuclear factor TOX was required for the development of all CD4 T lineages, indicating FMT and TSN might affect the immunity response in exposed mice as the expressions of *Tox* were decreased in the three exposures. This gene might be also served as a potential biomarker for FMT-ILI.

In conclusion, our results demonstrate that integrated analysis of the interaction of miRNA and mRNA is valuable to identify novel molecular mechanisms for TILI. More importantly, this study firstly provides evidence that the hepatic adaption to TILI existed after sustained TSN exposure and the mechanism may be related to the liver regeneration. Although the changes in miRNA and mRNA profiles need to be further confirmed by measuring the protein and metabolite levels, the findings from this study demonstrated that integrated miRNA-mRNA approach can be used as a tool to better understand the complex and dynamic behavior of TILI, which is also propitious to identify the pathological processes of HILI.

## Materials and Methods

### The extraction and fractionation of TSN

The fruit of *Melia toosendan* Sieb. et Zucc (FMT, ChuanLianZi in Chinese) was purchased from Zhejiang Chinese Medical University Medicine Plant (Hangzhou, China, product lot no. 130301) and authenticated by associated professor Liurong Chen, the botanist of College of Pharmaceutical Sciences, Zhejiang University, China. Fifty-kilogram dried FMT were soaked overnight in 95% ethanol (EtOH) and extracted with 95% EtOH (1:8, w/v) three times with 90 min for each time. The extracting solution was concentrated by evaporation under reduced pressure and then extracted with petroleum ether (PE) (1:1, v/v) for twice and subsequently by ethyl acetate (EtOAc) (1:1, v/v) for four times successively. The resulted ethyl acetate portion was subjected to column chromatography with 100 to 200 mesh silica gel and eluted with a gradient of petroleum ether and ethyl acetate to give the crude extract. The medium pressure octadecylsilyl (ODS) column was used to enrich the crude extract with setting elution time for 2 h and the methanol gradient concentration from 45–55%. High performance liquid chromatography (HPLC, Waters 2695, USA) was then performed to detect the fractions containing TSN, and these fractions were evaporated by reduced pressure and further purified by recrystallization. The resulted TSN was identified by comparing its retention time, UV spectra, and MS spectra with those of the standard (purity ≥ 98%, purchased from Shanghai yuanye Bio-Technology Co., Ltd., LOT number: 20130315) using HPLC-photodiode array (PDA)-mass spectrometry (MS, Thermo Finnigan LCQ DECA XP^Plus^, USA) analysis.

### Animals study

Male BALB/c mice (18–22 g, Silaike Co., Shanghai, China) were housed in an environmentally controlled room at 25 ± 1 °C with a relative humidity of 50 ± 10%, and a light-dark cycle of 12 h each. Food and tap water were provided *ad libitum*. Mice were allowed to acclimate for 3 days before the experiments were initiated. Then, the mice were randomly assigned to one of the following seven treatment groups (n = 8): vehicle control for 3 days, vehicle control for 9 days, vehicle control for 21 days, TSN at 80 mg/kg for 3 days, TSN at 80 mg/kg for 9 days, TSN at 80 mg/kg for 21 days, and TSN at 40 mg/kg for 9 days. The vehicle control groups were treated with 1% sodium carboxymethyl cellulose (1% CMC-Na) at 0.2 mL/10 g. The exposure doses and periods were determined by our preliminary experiments and the clinical dose of TSN for treating intestinal ascariasis^53^. In detail, the treatment dose of TSN used for intestinal ascariasis is 0.2 to 0.25 g daily for adults in clinic^53^ (i.e., 3.3 to 4.2 mg/kg daily for 60 kg adult), and the equivalent dose for mice is approximately 40 to 51 mg/kg daily based on body surface area^54^. Thus, we chose 40 mg/kg as the low dose. Moreover, based on the general toxicological results of our preliminary experiments, the high dose was decided to be 80 mg/kg as this dosage caused most serious liver toxicity after 9-days exposure, induced mild liver injury by 3-days treatment, and led to a recovery trend in liver toxicity after 21-days administration, while exposure to 40 mg/kg TSN for 9 days, the toxic effects were slight in mice. All the treatments were given at a frequency of intragastrical administration once a day for indicated days. The body weight of mice was recorded every day. Twenty-four hours after last administration, the mice were weighted and then sacrificed, and the blood samples were collected from orbital venous plexus and centrifuged at 4000 rpm for 15 min at 4 °C. The resulted serum samples were used for biochemical analysis. Tissues were collected as mentioned previously^21^,^22^. Briefly, slices with approximately 200 mg from the left lateral liver lobe from all exposures were snap-frozen in liquid nitrogen and stored at −80 °C for further microarray analysis. The remaining liver lobes were used for histopathological examinations. All the protocols and studies involving the animals were approved by the Animal Care and Use Committee of Zhejiang University School of Medicine and conducted in accordance with the guiding principles covered in the Use of Animals in Toxicology.

### Biochemical assay

The specific parameters in serum were examined using Cobas C8000 system (Roche Diagnostics, Germany) according to the manufacturer’s instructions with the appropriate kits, including ALT, AST, ALP, TBILI, TCHOL, and TG.

### Histopathological examinations

Livers were fixed immediately in 10% neutral-buffered formalin for at least 24 h, and then embedded in paraffin, sectioned into 4-μm thick slices, stained with hematoxylin and eosin, and finally evaluated by the pathologist in a blinded fashion with optical microscope.

### RNA extraction, purification, and quality assessment

The RNA extraction, purification, and quality assessment were performed as described previously^21^,^22^. Briefly, total RNA, including miRNA, was extracted and purified from frozen liver tissues using mirVana™ miRNA Isolation Kit (Ambion, TX, USA) in accordance with the manufacturer’s protocols. RNA quality was assessed with an Agilent 2100 Bioanalyzer and electrophoresis in 2% (v/v) agarose gels. Only RNAs with RNA integrity numbers (RINs) greater than 7.0 and 28S rRNA/18S rRNA more than 0.7 were used for microarray experiments.

### mRNA and miRNA microarray analysis

The mRNA expression profiles were obtained using the Affymetrix Mouse Genome 430 2.0 chips and Affymetrix products as mentioned in great detail in our previous study^55^. The Agilent Mouse miRNA V19.0 array containing 1,247 miRNAs was used for miRNA expression analysis. In total RNA, miRNA molecular was labeled using miRNA Complete Labeling and Hyb Kit (Agilent technologies, USA), hybridized in hybridization Oven (Agilent technologies, USA), and washed in staining dishes (Thermo Shandon, MA, USA) with Gene Expression Wash Buffer Kit (Agilent technologies, CA, USA), following the manufacturer’s instructions. Negative and positive controls are existed in these two arrays, in which the positive control transcripts were monitored to verify hybridization integrity, and the negative control features were set to estimate fluorescence background and background variance^56^,^57^,^58^.

### Microarray data analysis

mRNA gene expression data from the Affymetrix Mouse Genome 430 2.0 chips were input to the ArrayTrack^®^ v 3.1.5 for analysis. The normalization of the raw data and further analysis were conducted based on our previous studies^21^,^22^. Briefly, the raw microarray intensity data were normalized by MAS 5.0 algorithm and were further normalized per chip to the same median intensity value of 1000. Welch’s t-test within ArrayTrack^®^ was used to identify the DEGs between TSN and vehicle control groups with cutoffs of the *p* value < 0.05, absolute fold change > 2, and the mean channel intensity > 250. The cutoff for mean channel intensity was aimed to exclude false DEGs due to low abundance transcripts. The comparison was conducted at same treatment day of TSN and vehicle control groups. Then, the DEGs were further biologically interpreted using the Gene ontology (GO) database (www.geneontology.org/GO.doc.html) and Kyoto Encyclopedia of Genes and Genomes (KEGG) public pathway resource (www.genome.jp/kegg) within ArrayTrack^®^. PCA and HCA were conducted based on the global gene expression data using the auto-scaled method within ArrayTrack^®^. Venn diagram analysis was also performed on DEGs of the TSN-treated groups within ArrayTrack^®^.

For miRNA, the normalization of the raw data and further analysis were also performed based on previous studies^21^,^22^. For detail, the raw data were log2 transformed and normalized by Quantile algorithm within Gene Spring Software 11.0 (Agilent technologies, US). Only the mean intensity of miRNAs in at least one of all the groups > 4 (log2 transformed intensity) were considered, which was set to avoid false DEMs in next step due to low abundance features. Then, the student’s t-test in R software was performed to select the DEMs between TSN and vehicle control groups of the same treatment period. Only the miRNAs with *p* value less than 0.05 and an absolute fold change more than 1.5-fold between TSN and vehicle control groups were considered to be significantly changed. Finally, these DEMs related data of each group were input to the ArrayTrack^®^. PCA, HCA, and Venn diagram analysis were conducted to the treatments using the auto-scaled method within ArrayTrack^®^ based on these DEMs.

### mRNA and miRNA quantitation by qRT-PCR

To verify gene expression changes measured by microarray analysis, the qRT-PCR was carried out with the same RNA sample used for microarray. The detailed description was shown in our previous studies^21^,^22^. The sequences for the primers used for mRNA and miRNA were listed in Supplementary Tables S10 and S11. The quantitative real-time PCR was performed using the real-time thermal cycler (Mastercycler ep realplex 4, Eppendorf, USA) according to the manufacturer’s instructions. The method of 2^−ΔΔCt^ was used to generate the fold changes based on previous studies^21^,^22^.

### miRNA targets prediction

The DEMs were loaded into Ingenuity^®^ Pathway Analysis (IPA) (http://www.ingenuity.com) for their corresponding target mRNAs’ information as described previously^21^,^22^.

### Ingeniuty Pathway Analysis

The miRNA-mRNA intersection dataset was selected and analyzed based on our previous studies^21^,^22^. In more detail, the predicted target mRNAs that were also differentially expressed (*p* value < 0.05, absolute fold change >2) following exposure to TSN treatment were selected. Since miRNA mediated repression was mainly contributed to decreased mRNA abundance^59^, the target mRNAs changed in the opposite direction of miRNAs expression were generated as the miRNA-mRNA intersection dataset and analyzed by IPA systems. The biological and molecular functions of these genes were explored in IPA. The “tox analysis” function in IPA was performed to these genes for the “Molecular and Cellular Functions” and “Tox lists” analyses. A right-tailed fisher’s exact test was used to calculate the *p* value to determine the statistical significance of the biological functions or pathways within IPA.

### Statistical analysis

Statistical differences of body weight between the TSN and vehicle control groups were examined by unpaired and two-tailed Student’s t-test. The differences between these two groups of the data from serum biochemical parameter were determined by one-way ANOVA followed by Bonferroni Post Test in experiment 1 and were conducted by two-way ANOVA followed by Bonferroni Post Test in experiment 2. Values are represented as mean ± standard deviation (SD). A *p* value < 0.05 was considered to be significant.

## Additional Information

**How to cite this article**: Lu, X. *et al*. Integrated analysis of microRNA and mRNA expression profiles highlights the complex and dynamic behavior of toosendanin-induced liver injury in mice. *Sci. Rep.* **6**, 34225; doi: 10.1038/srep34225 (2016).

## Supplementary Material

Supplementary Information

## Figures and Tables

**Table 1:** miRNA-mRNA interactions.

	DEGs	DEMs	DEMs who have targets	miRNA-mRNA intersection dataset
TSN 80 mg/kg-3d	266	43	16	13 miRNA -25 target mRNA
TSN 80 mg/kg-9d	807	14	5	4 miRNA -45 target mRNA
TSN 80 mg/kg-21d	565	69	36	32 miRNA -190 target mRNA
