# Supplementary material for: Integrated analysis of microRNA and mRNA expression profiles highlights the complex and dynamic behavior of toosendanin-induced liver injury in mice
Source: Sci Rep. 2016 Oct 5;6:34225. doi: 10.1038/srep34225 (PMC5050432; doi:10.1038/srep34225)
Supplement: Supplementary Information [file srep34225-s1.pdf]

Integrated analysis of microRNA and mRNA expression profiles highlights the complex and dynamic behavior of toosendanin-induced liver injury in mice

Xiaoyan Lu[a], Cai Ji[a], Wei Tong[a], Xueping Lian[a], Ying Wu[a], Xiaohui Fan[a*], Yue Gao[b*]

[a]Pharmaceutical Informatics Institute, College of Pharmaceutical Sciences, Zhejiang University, Hangzhou 310058, China.

[b]Department of Pharmacology and Toxicology, Beijing Institute of Radiation Medicine, Beijing 100850, China.

*Correspondence should be addressed to Dr. Xiaohui Fan (E-mail:fanxh@zju.edu.cn, Tel/Fax: 86-579-88208596) or Dr. Yue Gao (E-mail: gaoyue@bmi.ac.cn, Tel/Fax: 86-10-6691312).

Supplementary Information

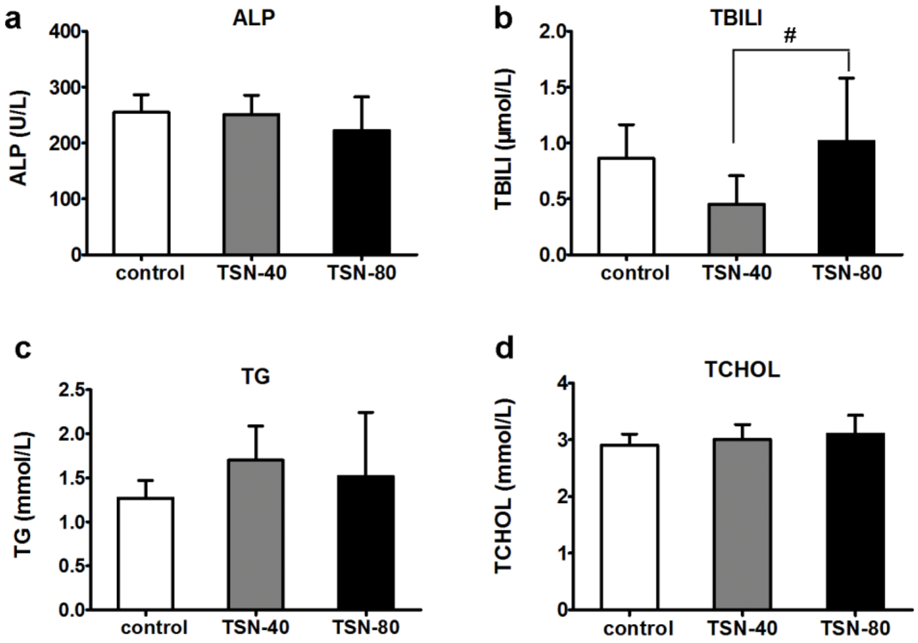

Supplementary Figure S1. The effects of 9-days 40 and 80 mg/kg TSN treatments on the selected serum biochemical parameters. (a) ALP, (b) TBILI, (c) TG, (d) TCHOL. TSN-40 indicates 40 mg/kg TSN treatment, and TSN-80 represents 80 mg/kg TSN exposure. $^{\#}p < 0.05$, compared between 40 and 80 mg/kg TSN treatments.

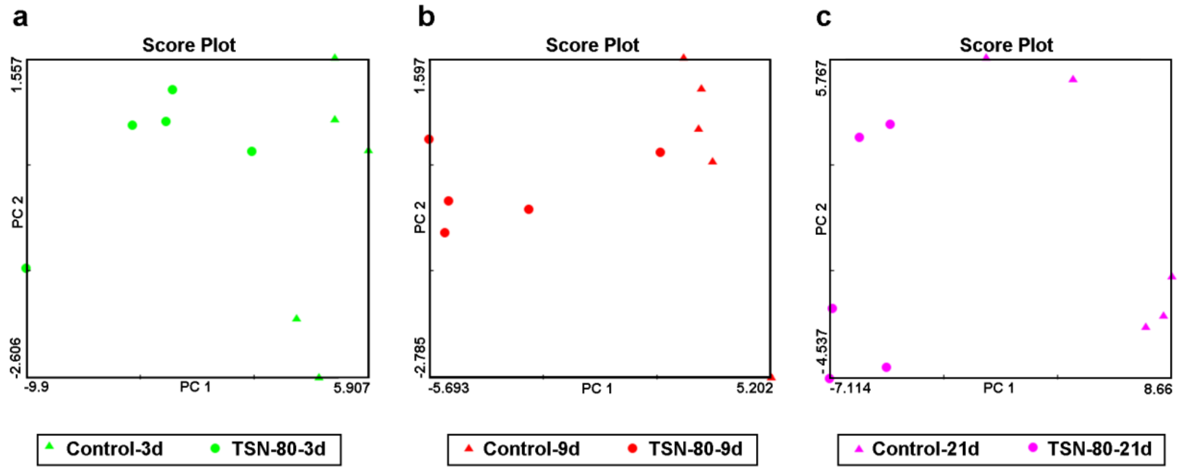

Supplementary Figure S2. The time-dependent effects of TSN on the miRNA expression profiles. (A-C) The PCA score plot of the liver samples from the mice exposure to 80 mg/kg TSN for 3 (a), 9(b), or 21(c) days based on the data of global miRNA expression profiles. TSN-80 represents 80 mg/kg TSN exposure.

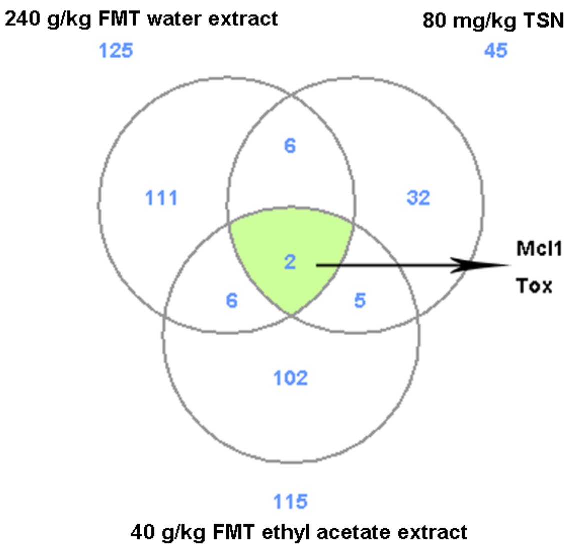

Supplementary Figure S3. The Venn diagram showed the common genes among the miRNA-mRNA intersections of 3-days 240 g/kg FMT water extract, 9-days 40 g/kg FMT ethyl acetate extract, and 9-days 80 mg/kg TSN exposures.

Supplementary Table S1. The overall numbers of DEGs and DEMs in 40 and 80 mg/kg TSN treatments compared with control group in the indicated day. The cutoff for selection of DEGs was based on a p value < 0.05 combined with absolute fold change (FC) > 2 and mean channel intensities > 250 by the Welch's t-test within Arraytrack®. The cutoff for selection of DEMs was according to a p value < 0.05 and FC > 1.5 by Student's t-test in R software. Before input miRNAs into R software, a filter which was only kept the probes expressed with mean intensity greater than 4 (log2 transformed intensity) in at least one group was used.

**Group**	**The number of DEGs**			**The number of DEMs**		
	**Total**	**Up-regulation**	**Down-Regulation**	**Total**	**Up-regulation**	**Down-Regulation**
TSN 80mg/kg 3d	266	214	52	43	29	14
TSN 80mg/kg 9d	807	595	212	14	10	4
TSN 80mg/kg 21d	565	371	194	69	22	47
TSN 40 mg/kg 9d	50	20	30	25	4	21

Supplementary Table S2. The common DEGs between 40 and 80 mg/kg TSN treatments for 9 days, which were identified by Manufacture_ID in Arraytrack®.

			Fold change	
Manufacture_ID	Gene name	LOCUSID	40 mg/kg	80 mg/kg
1425150_at	Acnat2	209186	2.9	11.5
1444518_at	Acox1	11430	-2.2	-2.5
1458442_at	AI132709	101494	3.9	26.0
1453588_at	Car3	12350	2.1	-7.8
1427422_at	EG624219	624219	3.1	2.3
1449525_at	Fmo3	14262	5.5	268.2
1436643_x_at	Hamp2	66438	2.1	2.7
1427229_at	Hmgcr	15357	2.0	4.4
1452388_at	Hspa1a	193740	-2.0	-6.0
1416630_at	Id3	15903	2.1	3.3
1426690_a_at	Srebf1	20787	2.4	2.3
1438129_at	Wsb2	59043	-3.8	-3.6

Supplementary Table S3. The common DEGs among the three time points of 80 mg/kg TSN treatments, which were identified by Manufacture_ID in Arraytrack®.

Manufacture_ID	Gene name	LOCUSID	Fold Change		
			3d	9d	21d
1430893_at	2610016E04Rik	100039008	-2.9	-55.9	-8.3
1419759_at	Abcb1a	18671	3.5	3.1	2.3
1419758_at	Abcb1a	18671	2.6	4.8	3.5
1456812_at	Abcd2	26874	4.4	9.0	5.7
1438431_at	Abcd2	26874	3.9	6.3	6.3
1419748_at	Abcd2	26874	5.2	7.4	4.7
1425150_at	Acnat2	209186	2.2	11.5	8.5
1422925_s_at	Acot3	171281	2.9	2.9	5.7
1423556_at	Akr1b7	11997	2.2	6.6	4.4
1418858_at	Aox3	71724	-2.1	-4.6	-2.2
1436504_x_at	Apoa4	11808	3.2	10.5	2.7
1417761_at	Apoa4	11808	3.9	6.5	3.4
1427513_at	BC024137	234757	-2.2	-6.4	-3.1
1451615_at	Ces8	234677	-4.3	-2.3	-3.3
1421363_at	Cyp2c39	13098	2.1	5.0	4.6
1419582_at	Cyp2c55	72082	-2.4	-2.5	2.8
1418780_at	Cyp39a1	56050	3.0	14.7	5.7
1424853_s_at	Cyp4a10 /// Cyp4a31	13117	3.4	9.0	7.9
1423257_at	Cyp4a14	13119	6.3	36.2	28.6
1417991_at	Dio1	13370	-2.4	-14.6	-6.0
1420722_at	Elovl3	12686	-3.3	-30.5	-8.8
1449525_at	Fmo3	14262	6.2	268.2	215.0
1421365_at	Fst	14313	2.6	3.5	3.6
1434458_at	Fst	14313	2.3	5.1	2.2
1420531_at	Hsd3b5	15496	-3.6	-204.1	-18.1
1452388_at	Hspa1a	193740	-4.3	-6.0	-8.2
1427127_x_at	Hspa1b	15511	-3.6	-7.9	-7.2
1452318_a_at	Hspa1b	15511	-3.7	-9.2	-7.0
1427126_at	Hspa1b	15511	-3.4	-7.8	-7.3
1436221_at	Ildr2	100039795	2.2	3.7	2.2
1416833_at	Keg1	64697	-2.3	-5.3	-4.0
1427512_a_at	Lama3	16774	-3.2	-10.5	-5.5
1427747_a_at	Lcn2	16819	7.4	13.0	5.9
1426808_at	Lgals3	16854	2.9	5.8	2.2
1436996_x_at	Lyz1	17110	2.1	2.2	2.4
1439426_x_at	Lyz1	17110	3.5	3.9	3.1

1428942_at	Mt2	17750	5.7	6.4	4.8
1430896_s_at	Nudt7	67528	-2.3	-9.3	-2.7
1431302_a_at	Nudt7	67528	-2.1	-7.2	-2.1
1420984_at	Pctp	18559	3.1	5.0	5.5
1452248_at	Plekhg5	269608	3.5	2.8	3.8
1436194_at	Prelid2	77619	2.0	3.4	2.1
1421430_at	Rad51l1	19363	4.6	14.8	11.7
1421425_a_at	Rcan2	53901	2.4	2.8	4.1
1437983_at	Sall1	58198	2.1	3.8	2.6
1421921_at	Serpina3m	20717	2.0	3.2	2.8
1429285_at	Serpina9	71907	-4.4	-17.5	-9.0
1420819_at	Sla	20491	2.7	3.0	2.8
1448502_at	Slc16a7	20503	2.0	2.4	2.0
1451460_a_at	Slc22a7	108114	-2.4	-10.8	-2.1
1447643_x_at	Snai2	20583	-2.2	3.0	7.0
1455899_x_at	Socs3	12702	4.5	2.1	-2.9
1456212_x_at	Socs3	12702	3.7	2.0	-2.1
1449816_at	Sult5a1	57429	-3.1	-9.1	-6.2
1446769_at	Ttc39c	72747	-2.3	-5.4	-3.3
1423397_at	Ugt2b38	100559	-5.3	-26.6	-12.3
1442537_at			-2.1	-3.2	-4.7

Supplementary Table S4. The 17 miRNAs differed significantly in the same manner between either two time points after 3, 9, and 21-days 80 mg/kg TSN exposures.

	Fold Change		
**microRNAs**	**3d**	**9d**	**21d**
mmu-miR-1892	-3.4	-2.1	
mmu-miR-802-5p		3.2	4.0
mmu-miR-6236		-1.5	-1.7
mmu-miR-677-3p	-1.6		-2.0
mmu-miR-378b	-1.6		-1.8
mmu-miR-30e-3p	-1.6		-1.8
mmu-miR-3474	5.2		4.4
mmu-miR-3473e	3.0		2.7
mmu-miR-3473b	4.4		3.7
mmu-miR-3473a	4.2		3.3
mmu-miR-3968	-1.9		-1.8
mmu-miR-3077-5p	1.9		2.0
mmu-miR-365-3p	-1.9		-2.1
mmu-miR-690	4.1		4.0

mmu-miR-5128	2.1	2.5
mmu-miR-126-5p	-1.6	-2.3
mmu-miR-100-5p	-1.6	-2.1

Supplementary Table S5.Validation of miRNA microarray results by qRT-PCR. Black bars indicate the mean fold change (±SD) derived from duplicate RT-PCR reactions. Gray bars indicate microarray data. NM stands for not measured.

	TSN-80-9d		TSN-40-9d		TSN-80-3d		TSN-80-21d	
**miRNAs**	**qRT-PCR**	**Microarray**	**qRT-PCR**	**Microarray**	**qRT-PCR**	**Microarray**	**qRT-PCR**	**Microarray**
miR-1927	1.8 ±0.7	2.2	1.4 ±0.3	1.6	NM	1.2	NM	-1.2
miR-802-5p	5.4 ±1.0	3.2	NM	1.7	NM	1.2	NM	4.0
miR-6236	-1.2 ±0.1	-1.5	NM	-1.1	NM	-1.3	NM	-1.7
miR-3968	NM	-1.4	NM	-1.1	-1.6 ±0.3	-1.9	1.7 ±0.5	-1.8
miR-126-5p	NM	1.0	NM	1.2	NM	-1.6	-2.1 ±0.9	-2.3
miR-100-5p	NM	-1.1	NM	1.2	-2.9 ±0.6	-1.6	-1.3 ±0.2	-2.1
miR-5100	NM	1.8	NM	1.0	NM	1.1	1.8 ±0.3	2.1
miR-3102-5p	NM	1.0	NM	-4.1	-3.9 ±0.9	-2.8	NM	1.1

Supplementary Table S6. The top five pathways of "Molecular and Cellular Functions" affected by 80 mg/kg TSN at each time point using "tox analysis" function in IPA. A right-tailed fisher's exact test was used to calculate the p-value to determine the statistical significance and a p-value < 0.05 was considered to be significant.

Time point	Molecular and Cellular Functions	*p* value	Molecules
3 days	Cellular Development	8.33E-06 - 3.72E-02	10
	Cellular Growth and Proliferation	8.33E-06 - 3.72E-02	9
	Cellular Function and Maintenance	3.05E-04 - 3.05E-02	7
	Cell Death and Survival	3.07E-04 - 3.67E-02	11
	Cell Cycle	6.75E-04 - 3.44E-02	6
9 days	Lipid Metabolism	3.89E-06 - 1.67E-02	13
	Small Molecule Biochemistry	3.89E-06 - 1.67E-02	16
	Molecular Transport	4.95E-06 - 1.67E-02	13
	Energy Production	3.23E-05 - 1.67E-02	9
	Cell-To-Cell Signaling and Interaction	4.32E-05 - 1.47E-02	10
21 days	Lipid Metabolism	9.13E-16 - 1.61E-03	62
	Molecular Transport	9.13E-16 - 1.61E-03	70

Small Molecule Biochemistry	9.13E-16 - 1.78E-03	70
Cellular Growth and Proliferation	1.43E-11 - 1.78E-03	91
Cell Death and Survival	6.95E-11 - 1.78E-03	87

Supplementary Table S7. The miRNA-mRNA intersection after 80 mg/kg TSN treatment for 3 days.

Name of miRNA	p-value	Fold Change	Source	Confidence	ID of mRNA	Symbol	p-value	Fold Change
mmu-miR-100-5p	2.96E-02	-1.6	TargetScan	High (predicted)	1447845_s_at	VNN1	1.30E-03	5.7
mmu-miR-139-5p	2.50E-02	-1.5	TargetScan	High (predicted)	1452232_at	GALNT7	5.70E-03	2.1
mmu-miR-139-5p	2.50E-02	-1.5	TargetScan	High (predicted)	1456389_at	ZEB2	1.50E-03	2.6
mmu-miR-3473a	9.63E-03	4.2	TargetScan	Moderate (predicted)	1450725_s_at	CA14	1.00E-04	-3.4
mmu-miR-3473a	9.63E-03	4.2	TargetScan	Moderate (predicted)	1451615_at	CES4A	2.38E-02	-4.3
mmu-miR-3473a	9.63E-03	4.2	TargetScan	Moderate (predicted)	1417991_at	DIO1	4.46E-02	-2.4
mmu-miR-188-5p	3.28E-02	2.1	TargetScan	Moderate (predicted)	1423555_a_at	IFI44	4.43E-02	-3.2
mmu-miR-3077-5p	1.72E-02	1.9	TargetScan	Moderate (predicted)	1446769_at	TTC39C	2.60E-03	-2.3
mmu-miR-3473b	6.53E-03	4.4	TargetScan	Moderate (predicted)	1451615_at	CES4A	2.38E-02	-4.3
mmu-miR-3473b	6.53E-03	4.4	TargetScan	Moderate (predicted)	1417991_at	DIO1	4.46E-02	-2.4
mmu-miR-365-3p	3.67E-03	-1.9	TargetScan	High (predicted)	1449360_at	CSF2RB	4.61E-02	5.2
mmu-miR-365-3p	3.67E-03	-1.9	TargetScan	Moderate (predicted)	1433657_at	FAM78A	3.67E-02	2.2
mmu-miR-365-3p	3.67E-03	-1.9	TargetScan	High (predicted)	1434142_at	PPM1D	1.14E-02	2.1
mmu-miR-365-3p	3.67E-03	-1.9	TargetScan	High (predicted)	1437983_at	SALL1	4.00E-03	2.1
mmu-miR-365-3p	3.67E-03	-1.9	TargetScan	High (predicted)	1416041_at	SGK1	0.00E00	2.1
mmu-miR-365-3p	3.67E-03	-1.9	TargetScan	Moderate (predicted)	1420928_at	ST6GAL1	1.30E-03	2.0
mmu-miR-370-3p	2.00E-02	1.9	TargetScan	Moderate (predicted)	1422095_a_at	CMPK2	1.00E-02	-4.0
mmu-miR-370-3p	2.00E-02	1.9	TargetScan	Moderate (predicted)	1436058_at	RSAD2	2.55E-02	-4.5
mmu-miR-5120	1.13E-02	3.0	TargetScan	Moderate (predicted)	1420722_at	ELOVL3	5.60E-03	-3.3
mmu-miR-5120	1.13E-02	3.0	TargetScan	Moderate (predicted)	1436058_at	RSAD2	2.55E-02	-4.5
mmu-miR-5120	1.13E-02	3.0	TargetScan	Moderate (predicted)	1443698_at	XAF1	3.53E-02	-2.1
mmu-miR-483-5p	4.78E-02	1.6	TargetScan	Moderate (predicted)	1449025_at	IFIT3	4.53E-02	-5.8
mmu-miR-494-3p	2.96E-02	3.0	TargetScan	Moderate (predicted)	1422095_a_at	CMPK2	1.00E-02	-4.0
mmu-miR-494-3p	2.96E-02	3.0	TargetScan	High (predicted)	1446769_at	TTC39C	2.60E-03	-2.3
mmu-miR-574-3p	3.29E-02	-3.9	TargetScan	Moderate (predicted)	1419647_a_at	IER3	8.20E-03	4.4

mmu-miR-3102-5p	1.27E-02	-2.8	TargetScan	Moderate (predicted)	1449363_at	ATF3	8.40E-03	2.4
mmu-miR-3102-5p	1.27E-02	-2.8	TargetScan	Moderate (predicted)	1433657_at	FAM78A	3.67E-02	2.2
mmu-miR-3102-5p	1.27E-02	-2.8	TargetScan	High (predicted)	1438945_x_at	GJA1	4.00E-04	2.0
mmu-miR-3102-5p	1.27E-02	-2.8	TargetScan	Moderate (predicted)	1423854_a_at	RASL11B	1.30E-03	2.1
mmu-miR-3102-5p	1.27E-02	-2.8	TargetScan	Moderate (predicted)	1427963_s_at	RDH16	1.90E-03	2.2
mmu-miR-3102-5p	1.27E-02	-2.8	TargetScan	Moderate (predicted)	1418571_at	TNFRSF12A	2.52E-02	2.9

Supplementary Table S8. The miRNA-mRNA intersection after 80 mg/kg TSN treatment for 9 days.

Name of miRNA	p-value	Fold Change	Source	Confidence	ID of mRNA	Symbol	p-value	Fold Change
mmu-miR-29b-3p	1.53E-02	1.8	TargetScan	Moderate (predicted)	1422815_at	C9	7.50E-03	-5.2
mmu-miR-29b-3p	1.53E-02	1.8	TargetScan	Moderate (predicted)	1438597_x_at	MORF4L1	6.00E-04	-2.4
mmu-miR-29b-3p	1.53E-02	1.8	TargetScan	High (predicted)	1436736_x_at	NREP	1.90E-02	-7.5
mmu-miR-29b-3p	1.53E-02	1.8	TargetScan	High (predicted)	1451460_a_at	SLC22A7	4.30E-03	-10.8
mmu-miR-29b-3p	1.53E-02	1.8	TargetScan	High (predicted)	1451418_a_at	SPSB4	2.70E-03	-3.8
mmu-miR-29b-3p	1.53E-02	1.8	TargetScan	High (predicted)	1447700_x_at	SS18L1	3.00E-04	-12.6
mmu-miR-320-3p	7.30E-03	-1.9	TargetScan	High (predicted)	1454918_at	AGPS	7.00E-04	2.2
mmu-miR-320-3p	7.30E-03	-1.9	TargetScan	High (predicted)	1430059_at	DCAF12	3.99E-02	2.0
mmu-miR-320-3p	7.30E-03	-1.9	TargetScan	High (predicted)	1457233_at	DNAJA2	5.70E-03	2.3
mmu-miR-320-3p	7.30E-03	-1.9	TargetScan	High (predicted)	1447685_x_at	ETS2	3.74E-02	3.1
mmu-miR-320-3p	7.30E-03	-1.9	TargetScan	High (predicted)	1450505_a_at	FAM134B	8.00E-04	3.6
mmu-miR-320-3p	7.30E-03	-1.9	TargetScan	Moderate (predicted)	1434301_at	FAM84B	3.00E-03	2.4
mmu-miR-320-3p	7.30E-03	-1.9	TargetScan	High (predicted)	1442873_at	FIGN	9.00E-04	2.7
mmu-miR-320-3p	7.30E-03	-1.9	TargetScan	High (predicted)	1438558_x_at	FOXQ1	1.31E-02	3.0
mmu-miR-320-3p	7.30E-03	-1.9	TargetScan	Moderate (predicted)	1431334_a_at	GSKIP	1.09E-02	2.4
mmu-miR-320-3p	7.30E-03	-1.9	TargetScan	High (predicted)	1455721_at	GSPT2	1.00E-03	3.1
mmu-miR-320-3p	7.30E-03	-1.9	TargetScan	Moderate (predicted)	1436756_x_at	HADH	1.44E-02	2.6
mmu-miR-320-3p	7.30E-03	-1.9	TargetScan	High (predicted)	1436221_at	ILDR2	1.00E-04	3.7

mmu-miR-320-3p	7.30E-03	-1.9	TargetScan	Moderate (predicted)	1456498_at	ITGA4	8.70E-03	2.1
mmu-miR-320-3p	7.30E-03	-1.9	TargetScan	High (predicted)	1456156_at	LEPR	4.83E-02	2.9
mmu-miR-320-3p	7.30E-03	-1.9	TargetScan	Moderate (predicted)	1431056_a_at	LPL	2.00E-04	2.7
mmu-miR-320-3p	7.30E-03	-1.9	miRecords	Experimentally Observed	1437527_x_at	MCL1	0.00E00	3.0
mmu-miR-320-3p	7.30E-03	-1.9	TargetScan	High (predicted)	1437627_at	MEX3D	8.80E-03	2.1
mmu-miR-320-3p	7.30E-03	-1.9	TargetScan	High (predicted)	1439696_at	NR2C2	2.33E-02	2.2
mmu-miR-320-3p	7.30E-03	-1.9	TargetScan	High (predicted)	1423372_at	POLE4	3.80E-03	2.1
mmu-miR-320-3p	7.30E-03	-1.9	TargetScan	High (predicted)	1451140_s_at	PRKAG2	0.00E00	2.4
mmu-miR-320-3p	7.30E-03	-1.9	TargetScan	High (predicted)	1424084_at	PTBP3	6.60E-03	2.0
mmu-miR-320-3p	7.30E-03	-1.9	TargetScan	High (predicted)	1455938_x_at	RAD21	2.00E-03	2.1
mmu-miR-320-3p	7.30E-03	-1.9	TargetScan	High (predicted)	1420502_at	SAT1	0.00E00	2.4
mmu-miR-320-3p	7.30E-03	-1.9	TargetScan	Moderate (predicted)	1417815_a_at	SERINC3	0.00E00	3.0
mmu-miR-320-3p	7.30E-03	-1.9	TargetScan	High (predicted)	1417116_at	SLC6A8	1.43E-02	2.1
mmu-miR-320-3p	7.30E-03	-1.9	TargetScan	Moderate (predicted)	1417695_a_at	SOAT1	9.00E-04	2.3
mmu-miR-320-3p	7.30E-03	-1.9	TargetScan	High (predicted)	1425536_at	STX3	4.00E-04	2.2
mmu-miR-320-3p	7.30E-03	-1.9	TargetScan	Moderate (predicted)	1455493_at	SYNE1	0.00E00	2.4
mmu-miR-320-3p	7.30E-03	-1.9	TargetScan	High (predicted)	1454018_at	TLK2	1.70E-02	2.2
mmu-miR-320-3p	7.30E-03	-1.9	TargetScan	High (predicted)	1430592_at	TMEM106B	1.94E-02	3.3
mmu-miR-320-3p	7.30E-03	-1.9	TargetScan	Moderate (predicted)	1442039_at	TOX	6.90E-03	7.8
mmu-miR-320-3p	7.30E-03	-1.9	TargetScan	Moderate (predicted)	1438051_at	TTC14	2.30E-03	2.1
mmu-miR-320-3p	7.30E-03	-1.9	TargetScan, miRecords	Experimentally Observed, High (predicted)	1438118_x_at	VIM	1.16E-02	2.3
mmu-miR-320-3p	7.30E-03	-1.9	TargetScan	High (predicted)	1434465_x_at	VLDLR	2.00E-04	3.7
mmu-miR-342-3p	4.62E-02	2.0	TargetScan	High (predicted)	1425107_a_at	LIFR	6.00E-04	-4.7
mmu-miR-500-3p	3.54E-02	1.7	TargetScan	Moderate (predicted)	1425898_x_at	OLFM3	0.00E00	-2.2
mmu-miR-500-3p	3.54E-02	1.7	TargetScan	Moderate (predicted)	1416432_at	PFKFB3	7.50E-03	-2.5
mmu-miR-500-3p	3.54E-02	1.7	TargetScan	High (predicted)	1449051_at	PPARA	2.90E-03	-2.0
mmu-miR-500-3p	3.54E-02	1.7	TargetScan	Moderate (predicted)	1421242_at	RNF144A	2.58E-02	-3.5

Supplementary Table S9. The miRNA-mRNA intersection after 80 mg/kg TSN treatment for 21 days.

Name of miRNA	p-value	Fold Change	Source	Confidence	ID of mRNA	Symbol	p-value	Fold Change
mmu-miR-98-5p	5.99E-03	-1.8	TargetScan, miRecords	Experimentally Observed,High (predicted)	1453289_at	AGO4	9.60E-03	2.0
mmu-miR-98-5p	5.99E-03	-1.8	TargetScan	High (predicted)	1418250_at	ARL4D	2.40E-03	2.6
mmu-miR-98-5p	5.99E-03	-1.8	TargetScan	High (predicted)	1429946_at	CCDC141	1.34E-02	2.1
mmu-miR-98-5p	5.99E-03	-1.8	Ingenuity Expert Findings,TarBase,TargetScan, miRecords	Experimentally Observed,High (predicted)	1417420_at	CCND1	6.00E-04	2.7
mmu-miR-98-5p	5.99E-03	-1.8	TargetScan	High (predicted)	1424638_at	CDKN1A	1.09E-02	6.9
mmu-miR-98-5p	5.99E-03	-1.8	TargetScan	High (predicted)	1434272_at	CPEB2	1.80E-03	2.0
mmu-miR-98-5p	5.99E-03	-1.8	TargetScan	Moderate (predicted)	1451453_at	DAPK2	3.00E-04	2.3
mmu-miR-98-5p	5.99E-03	-1.8	TargetScan	High (predicted)	1436545_at	DTX4	7.00E-04	2.2
mmu-miR-98-5p	5.99E-03	-1.8	TargetScan	High (predicted)	1450268_at	FIGN	4.40E-03	2.1
mmu-miR-98-5p	5.99E-03	-1.8	TargetScan	High (predicted)	1438169_a_at	FRMD4B	2.00E-04	3.6
mmu-miR-98-5p	5.99E-03	-1.8	TargetScan	High (predicted)	1455417_at	KCNJ11	4.95E-02	3.2
mmu-miR-98-5p	5.99E-03	-1.8	TargetScan	High (predicted)	1439038_at	KHNYN	4.62E-02	2.3
mmu-miR-98-5p	5.99E-03	-1.8	TargetScan	High (predicted)	1425434_a_at	MSR1	4.62E-02	2.0
mmu-miR-98-5p	5.99E-03	-1.8	TarBase, TargetScan, miRecords	Experimentally Observed,High (predicted)	1437457_a_at	MTPN	0.00E00	3.0
mmu-miR-98-5p	5.99E-03	-1.8	TargetScan	High (predicted)	1427938_at	MYCBP	1.00E-04	2.3
mmu-miR-98-5p	5.99E-03	-1.8	TargetScan	High (predicted)	1417155_at	MYCN	3.07E-02	4.7
mmu-miR-98-5p	5.99E-03	-1.8	TarBase	Experimentally Observed	1449061_a_at	PRIM1	4.57E-02	3.1
mmu-miR-98-5p	5.99E-03	-1.8	TargetScan	High (predicted)	1455265_a_at	RGS16	1.09E-02	11.3
mmu-miR-98-5p	5.99E-03	-1.8	TargetScan	Moderate (predicted)	1434628_a_at	RHPN2	0.00E00	3.4
mmu-miR-98-5p	5.99E-03	-1.8	TargetScan	High (predicted)	1419394_s_at	S100A8	3.85E-02	2.0
mmu-miR-98-5p	5.99E-03	-1.8	TargetScan	High (predicted)	1415965_at	SCD	2.00E-04	6.3
mmu-miR-98-5p	5.99E-03	-1.8	TarBase	Experimentally Observed	1416750_at	SIGMAR1	6.00E-04	2.0
mmu-miR-98-5p	5.99E-03	-1.8	TarBase, TargetScan	Experimentally Observed, High (predicted)	1456003_a_at	SLC1A4	1.00E-04	2.9
mmu-miR-98-5p	5.99E-03	-1.8	TargetScan	High (predicted)	1433699_at	TNFAIP3	5.00E-04	2.6
mmu-miR-98-5p	5.99E-03	-1.8	TargetScan	High (predicted)	1448666_s_at	TOB2	1.00E-04	3.5

mmu-miR-98-5p	5.99E-03	-1.8	TargetScan	High (predicted)	1434277_a_at	YPEL2	2.50E-03	2.0
mmu-miR-100-5p	5.67E-03	-2.1	TargetScan	Moderate (predicted)	1427938_at	MYCBP	1.00E-04	2.3
mmu-miR-100-5p	5.67E-03	-2.1	TargetScan	High (predicted)	1428372_at	ST5	3.00E-04	2.3
mmu-miR-100-5p	5.67E-03	-2.1	TargetScan	High (predicted)	1418486_at	VNN1	8.80E-03	2.5
mmu-miR-10a-5p	1.00E-02	-1.7	TargetScan	High (predicted)	1454617_at	ARRDC3	2.42E-02	2.3
mmu-miR-10a-5p	1.00E-02	-1.7	TargetScan	Moderate (predicted)	1434798_at	ATP6V0D2	1.50E-03	3.3
mmu-miR-10a-5p	1.00E-02	-1.7	TargetScan	High (predicted)	1450268_at	FIGN	4.40E-03	2.1
mmu-miR-10a-5p	1.00E-02	-1.7	TargetScan	Moderate (predicted)	1453587_at	GGT6	2.30E-03	2.1
mmu-miR-10a-5p	1.00E-02	-1.7	TargetScan	Moderate (predicted)	1417980_a_at	INSIG2	7.00E-04	2.9
mmu-miR-10a-5p	1.00E-02	-1.7	TargetScan	Moderate (predicted)	1443327_at	KIAA0319	3.90E-03	2.6
mmu-miR-10a-5p	1.00E-02	-1.7	TargetScan	High (predicted)	1451313_a_at	LGALSL	0.00E00	4.2
mmu-miR-10a-5p	1.00E-02	-1.7	TargetScan	Moderate (predicted)	1426913_at	LSS	3.60E-03	2.7
mmu-miR-10a-5p	1.00E-02	-1.7	TargetScan	High (predicted)	1425434_a_at	MSR1	4.62E-02	2.0
mmu-miR-10a-5p	1.00E-02	-1.7	TargetScan	High (predicted)	1427938_at	MYCBP	1.00E-04	2.3
mmu-miR-10a-5p	1.00E-02	-1.7	TargetScan	High (predicted),Moderate (predicted)	1421382_at	PRLR	1.00E-04	2.9
mmu-miR-10a-5p	1.00E-02	-1.7	TargetScan	Moderate (predicted)	1421411_at	PSTPIP2	4.50E-03	3.6
mmu-miR-10a-5p	1.00E-02	-1.7	TargetScan	Moderate (predicted)	1448754_at	RBP1	4.93E-02	2.4
mmu-miR-10a-5p	1.00E-02	-1.7	TargetScan	Moderate (predicted)	1451727_at	SLU7	1.71E-02	2.6
mmu-miR-10a-5p	1.00E-02	-1.7	TargetScan	Moderate (predicted)	1438855_x_at	TNFAIP2	2.00E-04	2.8
mmu-miR-125b-5p	6.76E-03	-1.6	TargetScan	High (predicted)	1443870_at	ABCC4	1.40E-03	3.4
mmu-miR-125b-5p	6.76E-03	-1.6	TargetScan	High (predicted)	1427052_at	ACACB	1.40E-02	3.7
mmu-miR-125b-5p	6.76E-03	-1.6	TargetScan	High (predicted)	1423315_at	BBC3	1.32E-02	2.2
mmu-miR-125b-5p	6.76E-03	-1.6	TargetScan	Moderate (predicted)	1428512_at	BHLHB9	2.43E-02	2.2
mmu-miR-125b-5p	6.76E-03	-1.6	TargetScan	High (predicted)	1436545_at	DTX4	7.00E-04	2.2
mmu-miR-125b-5p	6.76E-03	-1.6	TargetScan	Moderate (predicted)	1427229_at	HMGCR	4.80E-03	4.1
mmu-miR-125b-5p	6.76E-03	-1.6	TargetScan	High (predicted)	1439038_at	KHNYN	4.62E-02	2.3
mmu-miR-125b-5p	6.76E-03	-1.6	TargetScan	Moderate (predicted)	1446316_at	LPIN2	1.86E-02	3.5
mmu-miR-125b-5p	6.76E-03	-1.6	TargetScan	High (predicted)	1425673_at	LPP	5.00E-04	2.1

mmu-miR-125b-5p	6.76E-03	-1.6	TargetScan	High (predicted)	1437527_x_at	MCL1	3.80E-03	2.3
mmu-miR-125b-5p	6.76E-03	-1.6	TargetScan	Moderate (predicted)	1428167_a_at	MPZL1	1.48E-02	2.1
mmu-miR-125b-5p	6.76E-03	-1.6	TargetScan	Moderate (predicted)	1428547_at	NT5E	1.00E-04	3.3
mmu-miR-125b-5p	6.76E-03	-1.6	Ingenuity Expert Findings, TargetScan	Experimentally Observed,High (predicted)	1420984_at	PCTP	7.00E-04	5.5
mmu-miR-125b-5p	6.76E-03	-1.6	TargetScan	Moderate (predicted)	1419820_at	PKHD1	2.90E-03	2.5
mmu-miR-125b-5p	6.76E-03	-1.6	TargetScan	High (predicted)	1436194_at	PRELID2	2.30E-03	2.1
mmu-miR-125b-5p	6.76E-03	-1.6	TargetScan	High (predicted)	1447670_at	PSMD9	1.20E-03	2.6
mmu-miR-125b-5p	6.76E-03	-1.6	TargetScan	High (predicted)	1421411_at	PSTPIP2	4.50E-03	3.6
mmu-miR-125b-5p	6.76E-03	-1.6	Ingenuity Expert Findings, TargetScan	Experimentally Observed,High (predicted)	1415965_at	SCD	2.00E-04	6.3
mmu-miR-125b-5p	6.76E-03	-1.6	TargetScan	Moderate (predicted)	1426663_s_at	SLC45A3	5.00E-04	3.7
mmu-miR-125b-5p	6.76E-03	-1.6	TargetScan	High (predicted)	1420372_at	SNTB2	2.80E-03	2.0
mmu-miR-125b-5p	6.76E-03	-1.6	TargetScan	Moderate (predicted)	1438855_x_at	TNFAIP2	2.00E-04	2.8
mmu-miR-125b-5p	6.76E-03	-1.6	TargetScan	High (predicted)	1433699_at	TNFAIP3	5.00E-04	2.6
mmu-miR-126-3p	9.83E-04	-1.5	TarBase	Experimentally Observed	1426663_s_at	SLC45A3	5.00E-04	3.7
mmu-miR-126-5p	3.43E-04	-2.3	TarBase, miRecords	Experimentally Observed	1426663_s_at	SLC45A3	5.00E-04	3.7
mmu-miR-140-3p	5.83E-03	-1.7	TargetScan	High (predicted)	1454918_at	AGPS	0.00E00	2.2
mmu-miR-140-3p	5.83E-03	-1.7	TargetScan	High (predicted)	1459846_x_at	CNPY2	1.70E-03	2.2
mmu-miR-140-3p	5.83E-03	-1.7	TargetScan	Moderate (predicted)	1436545_at	DTX4	7.00E-04	2.2
mmu-miR-140-3p	5.83E-03	-1.7	TargetScan	Moderate (predicted)	1433446_at	HMGCS1	9.00E-04	2.7
mmu-miR-140-3p	5.83E-03	-1.7	TargetScan	Moderate (predicted)	1418918_at	IGFBP1	8.30E-03	12.4
mmu-miR-140-3p	5.83E-03	-1.7	TargetScan	Moderate (predicted)	1434853_x_at	MKRN1	1.00E-04	2.5
mmu-miR-140-3p	5.83E-03	-1.7	TargetScan	Moderate (predicted)	1455025_at	PAQR9	1.11E-02	2.1
mmu-miR-140-3p	5.83E-03	-1.7	TargetScan	Moderate (predicted)	1436590_at	PPP1R3B	3.47E-02	2.0
mmu-miR-140-3p	5.83E-03	-1.7	TargetScan	Moderate (predicted)	1424156_at	RBL1	4.34E-02	2.2
mmu-miR-140-3p	5.83E-03	-1.7	TargetScan	High (predicted)	1456003_a_at	SLC1A4	1.00E-04	2.9
mmu-miR-143-3p	8.08E-03	-1.8	TargetScan	High (predicted)	1443870_at	ABCC4	1.40E-03	3.4
mmu-miR-143-3p	8.08E-03	-1.8	TargetScan	High (predicted)	1423315_at	BBC3	1.32E-02	2.2
mmu-miR-143-3p	8.08E-03	-1.8	TargetScan	Moderate (predicted)	1448752_at	CA2	1.60E-03	2.1

mmu-miR-143-3p	8.08E-03	-1.8	TargetScan	High (predicted)	1434272_at	CPEB2	1.80E-03	2.0
mmu-miR-143-3p	8.08E-03	-1.8	TargetScan	High (predicted)	1455400_at	DDAH1	1.80E-03	3.2
mmu-miR-143-3p	8.08E-03	-1.8	TargetScan	High (predicted)	1435207_at	DIXDC1	9.00E-04	2.2
mmu-miR-143-3p	8.08E-03	-1.8	TargetScan	High (predicted)	1439264_x_at	LASP1	2.00E-04	5.8
mmu-miR-143-3p	8.08E-03	-1.8	TargetScan	High (predicted)	1425434_a_at	MSR1	4.62E-02	2.0
mmu-miR-143-3p	8.08E-03	-1.8	TargetScan	High (predicted)	1437457_a_at	MTPN	0.00E00	3.0
mmu-miR-143-3p	8.08E-03	-1.8	TargetScan	High (predicted)	1451727_at	SLU7	1.71E-02	2.6
mmu-miR-143-3p	8.08E-03	-1.8	TargetScan	Moderate (predicted)	1420447_at	SULT1E1	2.50E-03	53.0
mmu-miR-143-3p	8.08E-03	-1.8	TargetScan	High (predicted)	1460694_s_at	SVIL	3.00E-04	2.1
mmu-miR-143-3p	8.08E-03	-1.8	TargetScan	High (predicted)	1433473_x_at	TM2D2	0.00E00	2.3
mmu-miR-143-3p	8.08E-03	-1.8	TargetScan	High (predicted)	1448666_s_at	TOB2	1.00E-04	3.5
mmu-miR-145a-5p	1.71E-02	-2.1	TargetScan	Moderate (predicted)	1426392_a_at	ACTR3	0.00E00	2.3
mmu-miR-145a-5p	1.71E-02	-2.1	TargetScan	Moderate (predicted)	1456590_x_at	AKR1B1	0.00E00	2.5
mmu-miR-145a-5p	1.71E-02	-2.1	TargetScan	High (predicted)	1438143_s_at	ATXN2	0.00E00	2.2
mmu-miR-145a-5p	1.71E-02	-2.1	TargetScan	High (predicted)	1437455_a_at	BTG1	2.00E-04	2.3
mmu-miR-145a-5p	1.71E-02	-2.1	Ingenuity Expert Findings	Experimentally Observed	1422439_a_at	CDK4	1.50E-03	2.0
mmu-miR-145a-5p	1.71E-02	-2.1	TargetScan	High (predicted)	1418925_at	CELSR1	1.70E-03	3.0
mmu-miR-145a-5p	1.71E-02	-2.1	TargetScan	High (predicted)	1416332_at	CIRBP	6.50E-03	2.2
mmu-miR-145a-5p	1.71E-02	-2.1	TargetScan	Moderate (predicted)	1441326_at	CP	3.70E-03	2.5
mmu-miR-145a-5p	1.71E-02	-2.1	TargetScan	Moderate (predicted)	1436545_at	DTX4	7.00E-04	2.2
mmu-miR-145a-5p	1.71E-02	-2.1	TargetScan	Moderate (predicted)	1427229_at	HMGCR	4.80E-03	4.1
mmu-miR-145a-5p	1.71E-02	-2.1	TargetScan	High (predicted)	1439264_x_at	LASP1	2.00E-04	5.8
mmu-miR-145a-5p	1.71E-02	-2.1	TargetScan	High (predicted)	1446316_at	LPIN2	1.86E-02	3.5
mmu-miR-145a-5p	1.71E-02	-2.1	TargetScan	Moderate (predicted)	1444487_at	LRAT	3.66E-02	2.1
mmu-miR-145a-5p	1.71E-02	-2.1	TargetScan	High (predicted)	1428167_a_at	MPZL1	1.48E-02	2.1
mmu-miR-145a-5p	1.71E-02	-2.1	TargetScan	High (predicted)	1417155_at	MYCN	3.07E-02	4.7
mmu-miR-145a-5p	1.71E-02	-2.1	TargetScan	High (predicted)	1455025_at	PAQR9	1.11E-02	2.1
mmu-miR-145a-5p	1.71E-02	-2.1	TargetScan	High (predicted)	1437380_x_at	PGD	3.24E-02	2.2

mmu-miR-145a-5p	1.71E-02	-2.1	TargetScan	Moderate (predicted)	1421382_at	PRLR	1.00E-04	2.9
mmu-miR-145a-5p	1.71E-02	-2.1	TargetScan	High (predicted)	1421430_at	RAD51B	1.01E-02	11.7
mmu-miR-145a-5p	1.71E-02	-2.1	TargetScan	Moderate (predicted)	1456003_a_at	SLC1A4	1.00E-04	2.9
mmu-miR-145a-5p	1.71E-02	-2.1	TargetScan	High (predicted)	1426663_s_at	SLC45A3	5.00E-04	3.7
mmu-miR-145a-5p	1.71E-02	-2.1	TargetScan	High (predicted)	1420372_at	SNTB2	2.80E-03	2.0
mmu-miR-145a-5p	1.71E-02	-2.1	TargetScan	Moderate (predicted)	1438855_x_at	TNFAIP2	2.00E-04	2.8
mmu-miR-145a-5p	1.71E-02	-2.1	TargetScan	High (predicted)	1448147_at	TNFRSF19	4.90E-03	2.0
mmu-miR-151-5p	2.96E-03	-1.9	TargetScan	Moderate (predicted)	1416411_at	GSTM1	2.70E-03	2.0
mmu-miR-151-5p	2.96E-03	-1.9	TargetScan	Moderate (predicted)	1459841_x_at	LAPTM5	4.37E-02	2.1
mmu-miR-151-5p	2.96E-03	-1.9	TargetScan	Moderate (predicted)	1438974_x_at	PITPNM1	4.16E-02	2.2
mmu-miR-151-5p	2.96E-03	-1.9	TargetScan	Moderate (predicted)	1436590_at	PPP1R3B	3.47E-02	2.0
mmu-miR-151-5p	2.96E-03	-1.9	TargetScan	Moderate (predicted)	1421382_at	PRLR	1.00E-04	2.9
mmu-miR-151-5p	2.96E-03	-1.9	TargetScan	Moderate (predicted)	1438855_x_at	TNFAIP2	2.00E-04	2.8
mmu-miR-195a-5p	8.41E-03	-1.9	TargetScan	High (predicted)	1434185_at	ACACA	8.50E-03	2.5
mmu-miR-195a-5p	8.41E-03	-1.9	TargetScan	Moderate (predicted)	1427052_at	ACACB	1.40E-02	3.7
mmu-miR-195a-5p	8.41E-03	-1.9	TargetScan	High (predicted)	1453289_at	AGO4	9.60E-03	2.0
mmu-miR-195a-5p	8.41E-03	-1.9	TargetScan	High (predicted)	1447606_x_at	AQP11	0.00E00	2.7
mmu-miR-195a-5p	8.41E-03	-1.9	TargetScan	Moderate (predicted)	1435597_at	ATAD5	8.30E-03	2.0
mmu-miR-195a-5p	8.41E-03	-1.9	TargetScan	High (predicted)	1438143_s_at	ATXN2	0.00E00	2.2
mmu-miR-195a-5p	8.41E-03	-1.9	TargetScan	Moderate (predicted)	1448752_at	CA2	1.60E-03	2.1
mmu-miR-195a-5p	8.41E-03	-1.9	Ingenuity Expert Findings, TargetScan, miRecords	Experimentally Observed, High (predicted)	1417420_at	CCND1	6.00E-04	2.7
mmu-miR-195a-5p	8.41E-03	-1.9	TargetScan	High (predicted)	1430997_at	CD47	1.30E-03	2.5
mmu-miR-195a-5p	8.41E-03	-1.9	TargetScan	High (predicted)	1451306_at	CDCA7L	2.14E-02	2.4
mmu-miR-195a-5p	8.41E-03	-1.9	TargetScan	High (predicted)	1434272_at	CPEB2	1.80E-03	2.0
mmu-miR-195a-5p	8.41E-03	-1.9	TargetScan	High (predicted)	1435207_at	DIXDC1	9.00E-04	2.2
mmu-miR-195a-5p	8.41E-03	-1.9	TargetScan	High (predicted)	1423828_at	FASN	1.74E-02	3.9
mmu-miR-195a-5p	8.41E-03	-1.9	TargetScan	Moderate (predicted)	1448354_at	G6PD	6.00E-04	2.4
mmu-miR-195a-5p	8.41E-03	-1.9	TargetScan	Experimentally Observed	1419499_at	GPAM	1.50E-02	2.8

mmu-miR-195a-5p	8.41E-03	-1.9	TargetScan	High (predicted)	1436293_x_at	ILDR2	6.00E-04	2.2
mmu-miR-195a-5p	8.41E-03	-1.9	TargetScan	High (predicted)	1455417_at	KCNJ11	4.95E-02	3.2
mmu-miR-195a-5p	8.41E-03	-1.9	TargetScan	High (predicted)	1439038_at	KHNYN	4.62E-02	2.3
mmu-miR-195a-5p	8.41E-03	-1.9	TargetScan	High (predicted)	1425673_at	LPP	5.00E-04	2.1
mmu-miR-195a-5p	8.41E-03	-1.9	miRecords	Experimentally Observed	1437527_x_at	MCL1	3.80E-03	2.3
mmu-miR-195a-5p	8.41E-03	-1.9	TargetScan	High (predicted)	1417273_at	PDK4	3.50E-03	2.5
mmu-miR-195a-5p	8.41E-03	-1.9	miRecords	Experimentally Observed	1449061_a_at	PRIM1	4.57E-02	3.1
mmu-miR-195a-5p	8.41E-03	-1.9	TargetScan	Moderate (predicted)	1434628_a_at	RHPN2	0.00E00	3.4
mmu-miR-195a-5p	8.41E-03	-1.9	TarBase, TargetScan	Experimentally Observed,High (predicted)	1452649_at	RTN4	0.00E00	2.1
mmu-miR-195a-5p	8.41E-03	-1.9	TargetScan	High (predicted)	1437983_at	SALL1	4.10E-03	2.6
mmu-miR-195a-5p	8.41E-03	-1.9	TargetScan	High (predicted)	1420372_at	SNTB2	2.80E-03	2.0
mmu-miR-195a-5p	8.41E-03	-1.9	TargetScan	Moderate (predicted)	1451006_at	XDH	1.00E-04	2.8
mmu-miR-93-5p	4.92E-02	-1.5	TargetScan	High (predicted)	1424250_a_at	ARHGEF3	2.00E-04	2.4
mmu-miR-93-5p	4.92E-02	-1.5	TargetScan	High (predicted)	1430544_at	C2CD2	6.00E-04	2.2
mmu-miR-93-5p	4.92E-02	-1.5	TargetScan, miRecords	Experimentally Observed, High (predicted)	1417420_at	CCND1	6.00E-04	2.7
mmu-miR-93-5p	4.92E-02	-1.5	Ingenuity Expert Findings, TarBase, TargetScan, miRecords	Experimentally Observed,High (predicted)	1424638_at	CDKN1A	1.09E-02	6.9
mmu-miR-93-5p	4.92E-02	-1.5	TargetScan	High (predicted)	1423620_at	CENPQ	4.16E-02	2.2
mmu-miR-93-5p	4.92E-02	-1.5	TargetScan	High (predicted)	1451453_at	DAPK2	3.00E-04	2.3
mmu-miR-93-5p	4.92E-02	-1.5	TargetScan	High (predicted)	1438169_a_at	FRMD4B	2.00E-04	3.6
mmu-miR-93-5p	4.92E-02	-1.5	TargetScan	High (predicted)	1426834_s_at	KIAA0922	1.40E-03	2.0
mmu-miR-93-5p	4.92E-02	-1.5	TargetScan	High (predicted)	1439264_x_at	LASP1	2.00E-04	5.8
mmu-miR-93-5p	4.92E-02	-1.5	TargetScan	High (predicted)	1437527_x_at	MCL1	3.80E-03	2.3
mmu-miR-93-5p	4.92E-02	-1.5	TargetScan	High (predicted)	1449705_x_at	MCM3	4.78E-02	3.2
mmu-miR-93-5p	4.92E-02	-1.5	TargetScan	High (predicted)	1434853_x_at	MKRN1	1.00E-04	2.5
mmu-miR-93-5p	4.92E-02	-1.5	TargetScan	High (predicted)	1417155_at	MYCN	3.07E-02	4.7
mmu-miR-93-5p	4.92E-02	-1.5	TargetScan	Moderate (predicted)	1419820_at	PKHD1	2.90E-03	2.5
mmu-miR-93-5p	4.92E-02	-1.5	TargetScan	High (predicted)	1436590_at	PPP1R3B	3.47E-02	2.0
mmu-miR-93-5p	4.92E-02	-1.5	TargetScan	High (predicted)	1424156_at	RBL1	4.34E-02	2.2

mmu-miR-93-5p	4.92E-02	-1.5	TargetScan	High (predicted)	1437983_at	SALL1	4.10E-03	2.6
mmu-miR-93-5p	4.92E-02	-1.5	TargetScan	High (predicted)	1420372_at	SNTB2	2.80E-03	2.0
mmu-miR-93-5p	4.92E-02	-1.5	TargetScan	Moderate (predicted)	1433473_x_at	TM2D2	0.00E00	2.3
mmu-miR-93-5p	4.92E-02	-1.5	TargetScan	High (predicted)	1433699_at	TNFAIP3	5.00E-04	2.6
mmu-miR-93-5p	4.92E-02	-1.5	TargetScan	High (predicted)	1434465_x_at	VLDLR	8.00E-04	4.2
mmu-miR-93-5p	4.92E-02	-1.5	TargetScan	High (predicted)	1434277_a_at	YPEL2	2.50E-03	2.0
mmu-miR-3473a	1.89E-02	3.3	TargetScan	Moderate (predicted)	1433771_at	ABHD17B	1.30E-02	-2.3
mmu-miR-3473a	1.89E-02	3.3	TargetScan	Moderate (predicted)	1427251_at	ATP2A2	2.00E-04	-2.2
mmu-miR-3473a	1.89E-02	3.3	TargetScan	Moderate (predicted)	1435929_at	C12orf5	2.50E-03	-2.2
mmu-miR-3473a	1.89E-02	3.3	TargetScan	Moderate (predicted)	1451615_at	CES4A	1.40E-03	-3.3
mmu-miR-3473a	1.89E-02	3.3	TargetScan	Moderate (predicted)	1417991_at	DIO1	1.78E-02	-6.0
mmu-miR-3473a	1.89E-02	3.3	TargetScan	Moderate (predicted)	1422904_at	FMO2	1.00E-04	-3.1
mmu-miR-3473a	1.89E-02	3.3	TargetScan	High (predicted)	1424868_at	GLYAT	2.00E-04	-2.0
mmu-miR-3473a	1.89E-02	3.3	TargetScan	Moderate (predicted)	1426645_at	HSP90AA1	1.40E-03	-3.0
mmu-miR-3473a	1.89E-02	3.3	TargetScan	High (predicted)	1438274_at	IKZF4	7.20E-03	-6.2
mmu-miR-3473a	1.89E-02	3.3	TargetScan	Moderate (predicted)	1453276_at	KIF13B	1.10E-03	-2.7
mmu-miR-3473a	1.89E-02	3.3	TargetScan	High (predicted)	1459617_at	MAPK14	6.30E-03	-2.2
mmu-miR-3473a	1.89E-02	3.3	TargetScan	Moderate (predicted)	1457198_at	NRP1	4.46E-02	-2.5
mmu-miR-193a-3p	9.90E-03	-1.6	TargetScan	Moderate (predicted)	1418603_at	AVPR1A	1.00E-03	2.4
mmu-miR-193a-3p	9.90E-03	-1.6	Ingenuity Expert Findings, TargetScan	Experimentally Observed, High (predicted)	1417420_at	CCND1	6.00E-04	2.7
mmu-miR-193a-3p	9.90E-03	-1.6	TargetScan	Moderate (predicted)	1455400_at	DDAH1	1.80E-03	3.2
mmu-miR-193a-3p	9.90E-03	-1.6	TargetScan	High (predicted)	1416855_at	GAS1	2.65E-02	2.1
mmu-miR-193a-3p	9.90E-03	-1.6	TargetScan	High (predicted)	1425673_at	LPP	5.00E-04	2.1
mmu-miR-193a-3p	9.90E-03	-1.6	Ingenuity Expert Findings, TargetScan, miRecords	Experimentally Observed,High (predicted)	1437527_x_at	MCL1	3.80E-03	2.3
mmu-miR-193a-3p	9.90E-03	-1.6	TargetScan	High (predicted)	1421977_at	MMP19	1.60E-03	2.0
mmu-miR-193a-3p	9.90E-03	-1.6	TargetScan	High (predicted)	1417155_at	MYCN	3.07E-02	4.7
mmu-miR-193a-3p	9.90E-03	-1.6	TargetScan	High (predicted)	1428547_at	NT5E	1.00E-04	3.3
mmu-miR-193a-3p	9.90E-03	-1.6	TargetScan	Moderate (predicted)	1421382_at	PRLR	1.00E-04	2.9

mmu-miR-193a-3p	9.90E-03	-1.6	TargetScan	Moderate (predicted)	1451681_at	RDH16	3.00E-04	3.7
mmu-miR-193a-3p	9.90E-03	-1.6	TargetScan	Moderate (predicted)	1428372_at	ST5	3.00E-04	2.3
mmu-miR-199a-5p	2.20E-02	-1.7	TargetScan	High (predicted)	1434185_at	ACACA	8.50E-03	2.5
mmu-miR-199a-5p	2.20E-02	-1.7	TargetScan	Moderate (predicted)	1454918_at	AGPS	0.00E00	2.2
mmu-miR-199a-5p	2.20E-02	-1.7	TargetScan	High (predicted)	1447606_x_at	AQP11	0.00E00	2.7
mmu-miR-199a-5p	2.20E-02	-1.7	TargetScan	High (predicted)	1437455_a_at	BTG1	2.00E-04	2.3
mmu-miR-199a-5p	2.20E-02	-1.7	TargetScan	High (predicted)	1451306_at	CDCA7L	2.14E-02	2.4
mmu-miR-199a-5p	2.20E-02	-1.7	TargetScan	High (predicted)	1418925_at	CELSR1	1.70E-03	3.0
mmu-miR-199a-5p	2.20E-02	-1.7	TargetScan	High (predicted)	1422533_at	CYP51A1	1.80E-03	2.7
mmu-miR-199a-5p	2.20E-02	-1.7	TargetScan	High (predicted)	1418918_at	IGFBP1	8.30E-03	12.4
mmu-miR-199a-5p	2.20E-02	-1.7	TargetScan	High (predicted)	1455025_at	PAQR9	1.11E-02	2.1
mmu-miR-199a-5p	2.20E-02	-1.7	TargetScan	Moderate (predicted)	1455265_a_at	RGS16	1.09E-02	11.3
mmu-miR-199a-5p	2.20E-02	-1.7	TargetScan	High (predicted)	1426663_s_at	SLC45A3	5.00E-04	3.7
mmu-miR-19b-3p	6.44E-04	-1.6	TargetScan	High (predicted)	1428386_at	ACSL3	2.20E-03	4.9
mmu-miR-19b-3p	6.44E-04	-1.6	TargetScan	High (predicted)	1454617_at	ARRDC3	2.42E-02	2.3
mmu-miR-19b-3p	6.44E-04	-1.6	TargetScan	High (predicted)	1437455_a_at	BTG1	2.00E-04	2.3
mmu-miR-19b-3p	6.44E-04	-1.6	TargetScan, miRecords	Experimentally Observed, High (predicted)	1417420_at	CCND1	6.00E-04	2.7
mmu-miR-19b-3p	6.44E-04	-1.6	TargetScan	High (predicted)	1418925_at	CELSR1	1.70E-03	3.0
mmu-miR-19b-3p	6.44E-04	-1.6	TargetScan	High (predicted)	1422578_at	CS	1.00E-04	2.0
mmu-miR-19b-3p	6.44E-04	-1.6	TargetScan	High (predicted)	1420965_a_at	ENC1	0.00E00	3.1
mmu-miR-19b-3p	6.44E-04	-1.6	TargetScan	High (predicted)	1454671_at	INSIG1	4.00E-04	2.2
mmu-miR-19b-3p	6.44E-04	-1.6	TargetScan	High (predicted)	1417980_a_at	INSIG2	7.00E-04	2.9
mmu-miR-19b-3p	6.44E-04	-1.6	TargetScan	High (predicted)	1451313_a_at	LGALSL	0.00E00	4.2
mmu-miR-19b-3p	6.44E-04	-1.6	TargetScan	High (predicted)	1425673_at	LPP	5.00E-04	2.1
mmu-miR-19b-3p	6.44E-04	-1.6	TargetScan	Moderate (predicted)	1426913_at	LSS	3.60E-03	2.7
mmu-miR-19b-3p	6.44E-04	-1.6	TargetScan	High (predicted)	1428167_a_at	MPZL1	1.48E-02	2.1
mmu-miR-19b-3p	6.44E-04	-1.6	TargetScan	High (predicted)	1417155_at	MYCN	3.07E-02	4.7
mmu-miR-19b-3p	6.44E-04	-1.6	TargetScan	High (predicted)	1419820_at	PKHD1	2.90E-03	2.5

mmu-miR-19b-3p	6.44E-04	-1.6	TargetScan	Moderate (predicted)	1449310_at	PTGER2	8.20E-03	2.1
mmu-miR-19b-3p	6.44E-04	-1.6	TargetScan	Moderate (predicted)	1421430_at	RAD51B	1.01E-02	11.7
mmu-miR-19b-3p	6.44E-04	-1.6	TargetScan	High (predicted)	1418738_at	SCN1B	3.64E-02	2.6
mmu-miR-19b-3p	6.44E-04	-1.6	TargetScan	High (predicted)	1433699_at	TNFAIP3	5.00E-04	2.6
mmu-miR-21a-5p	1.31E-02	-1.6	TargetScan	High (predicted)	1438431_at	ABCD2	1.00E-04	6.3
mmu-miR-21a-5p	1.31E-02	-1.6	TargetScan	High (predicted)	1453289_at	AGO4	9.60E-03	2.0
mmu-miR-21a-5p	1.31E-02	-1.6	TargetScan	Moderate (predicted)	1430997_at	CD47	1.30E-03	2.5
mmu-miR-21a-5p	1.31E-02	-1.6	miRecords	Experimentally Observed	1424638_at	CDKN1A	1.09E-02	6.9
mmu-miR-21a-5p	1.31E-02	-1.6	TargetScan	Moderate (predicted)	1423620_at	CENPQ	4.16E-02	2.2
mmu-miR-21a-5p	1.31E-02	-1.6	TargetScan	Moderate (predicted)	1426064_at	CYP3A5	1.02E-02	2.6
mmu-miR-21a-5p	1.31E-02	-1.6	TargetScan	Moderate (predicted)	1444487_at	LRAT	3.66E-02	2.1
mmu-miR-21a-5p	1.31E-02	-1.6	TargetScan	Moderate (predicted)	1428547_at	NT5E	1.00E-04	3.3
mmu-miR-21a-5p	1.31E-02	-1.6	TargetScan	High (predicted)	1436590_at	PPP1R3B	3.47E-02	2.0
mmu-miR-21a-5p	1.31E-02	-1.6	TargetScan	Moderate (predicted)	1418760_at	RDH11	1.60E-03	3.2
mmu-miR-21a-5p	1.31E-02	-1.6	TargetScan	Moderate (predicted)	1452649_at	RTN4	0.00E00	2.1
mmu-miR-21a-5p	1.31E-02	-1.6	TargetScan	High (predicted)	1434520_at	SC5D	8.90E-03	2.6
mmu-miR-21a-5p	1.31E-02	-1.6	TargetScan	High (predicted)	1439934_at	SLC30A10	5.10E-03	2.3
mmu-miR-21a-5p	1.31E-02	-1.6	TargetScan	High (predicted)	1420372_at	SNTB2	2.80E-03	2.0
mmu-miR-27b-3p	2.08E-02	-1.6	TargetScan	Moderate (predicted)	1443870_at	ABCC4	1.40E-03	3.4
mmu-miR-27b-3p	2.08E-02	-1.6	TargetScan	Moderate (predicted)	1434185_at	ACACA	8.50E-03	2.5
mmu-miR-27b-3p	2.08E-02	-1.6	TargetScan	High (predicted)	1454918_at	AGPS	0.00E00	2.2
mmu-miR-27b-3p	2.08E-02	-1.6	TargetScan	High (predicted)	1447606_x_at	AQP11	0.00E00	2.7
mmu-miR-27b-3p	2.08E-02	-1.6	TargetScan	Moderate (predicted)	1418250_at	ARL4D	2.40E-03	2.6
mmu-miR-27b-3p	2.08E-02	-1.6	TargetScan	High (predicted)	1423315_at	BBC3	1.32E-02	2.2
mmu-miR-27b-3p	2.08E-02	-1.6	TargetScan	High (predicted)	1437455_a_at	BTG1	2.00E-04	2.3
mmu-miR-27b-3p	2.08E-02	-1.6	TargetScan	High (predicted)	1430544_at	C2CD2	6.00E-04	2.2
mmu-miR-27b-3p	2.08E-02	-1.6	TargetScan	High (predicted)	1418780_at	CYP39A1	4.00E-04	5.7
mmu-miR-27b-3p	2.08E-02	-1.6	TargetScan	High (predicted)	1455400_at	DDAH1	1.80E-03	3.2

mmu-miR-27b-3p	2.08E-02	-1.6	TargetScan	High (predicted)	1436545_at	DTX4	7.00E-04	2.2
mmu-miR-27b-3p	2.08E-02	-1.6	TargetScan	High (predicted)	1420965_a_at	ENC1	0.00E00	3.1
mmu-miR-27b-3p	2.08E-02	-1.6	TargetScan	High (predicted)	1419499_at	GPAM	1.50E-02	2.8
mmu-miR-27b-3p	2.08E-02	-1.6	TargetScan	Moderate (predicted)	1422892_s_at	HLA-DRA	7.70E-03	2.2
mmu-miR-27b-3p	2.08E-02	-1.6	TargetScan	High (predicted)	1427229_at	HMGCR	4.80E-03	4.1
mmu-miR-27b-3p	2.08E-02	-1.6	TargetScan	High (predicted)	1433446_at	HMGCS1	9.00E-04	2.7
mmu-miR-27b-3p	2.08E-02	-1.6	TargetScan	High (predicted)	1439264_x_at	LASP1	2.00E-04	5.8
mmu-miR-27b-3p	2.08E-02	-1.6	TargetScan	High (predicted)	1446316_at	LPIN2	1.86E-02	3.5
mmu-miR-27b-3p	2.08E-02	-1.6	TargetScan	High (predicted)	1425673_at	LPP	5.00E-04	2.1
mmu-miR-27b-3p	2.08E-02	-1.6	TargetScan	High (predicted)	1428223_at	MFSD2A	1.06E-02	2.6
mmu-miR-27b-3p	2.08E-02	-1.6	TargetScan	High (predicted)	1427938_at	MYCBP	1.00E-04	2.3
mmu-miR-27b-3p	2.08E-02	-1.6	TargetScan	High (predicted)	1454903_at	NGFR	2.60E-03	2.1
mmu-miR-27b-3p	2.08E-02	-1.6	TargetScan	High (predicted)	1455025_at	PAQR9	1.11E-02	2.1
mmu-miR-27b-3p	2.08E-02	-1.6	TargetScan	High (predicted)	1417273_at	PDK4	3.50E-03	2.5
mmu-miR-27b-3p	2.08E-02	-1.6	TargetScan	High (predicted)	1417689_a_at	PDZK1IP1	7.00E-04	2.9
mmu-miR-27b-3p	2.08E-02	-1.6	TargetScan	Moderate (predicted)	1421382_at	PRLR	1.00E-04	2.9
mmu-miR-27b-3p	2.08E-02	-1.6	TargetScan	Moderate (predicted)	1453578_at	PTER	6.00E-04	2.2
mmu-miR-27b-3p	2.08E-02	-1.6	TargetScan	High (predicted)	1421425_a_at	RCAN2	0.00E00	4.1
mmu-miR-27b-3p	2.08E-02	-1.6	TargetScan	High (predicted)	1451727_at	SLU7	1.71E-02	2.6
mmu-miR-27b-3p	2.08E-02	-1.6	TargetScan	High (predicted)	1440311_at	SORBS1	5.40E-03	2.3
mmu-miR-27b-3p	2.08E-02	-1.6	TargetScan	High (predicted)	1425484_at	TOX	2.72E-02	2.7
mmu-miR-27b-3p	2.08E-02	-1.6	TargetScan	High (predicted)	1434277_a_at	YPEL2	2.50E-03	2.0
mmu-miR-3077-5p	2.07E-02	2.0	TargetScan	Moderate (predicted)	1416432_at	PFKFB3	4.00E-04	-2.9
mmu-miR-3077-5p	2.07E-02	2.0	TargetScan	Moderate (predicted)	1446769_at	TTC39C	2.30E-02	-3.3
mmu-miR-378d	6.76E-03	-1.6	TargetScan	Moderate (predicted)	1424250_a_at	ARHGEF3	2.00E-04	2.4
mmu-miR-378d	6.76E-03	-1.6	TargetScan	Moderate (predicted)	1424143_a_at	CDT1	4.05E-02	3.2
mmu-miR-378d	6.76E-03	-1.6	TargetScan	Moderate (predicted)	1435207_at	DIXDC1	9.00E-04	2.2
mmu-miR-378d	6.76E-03	-1.6	TargetScan	Moderate (predicted)	1417880_at	G6PC	1.84E-02	2.3

mmu-miR-378d	6.76E-03	-1.6	TargetScan	Moderate (predicted)	1425673_at	LPP	5.00E-04	2.1
mmu-miR-378d	6.76E-03	-1.6	TargetScan	Moderate (predicted)	1428547_at	NT5E	1.00E-04	3.3
mmu-miR-378d	6.76E-03	-1.6	TargetScan	Moderate (predicted)	1450516_a_at	RAB17	2.23E-02	3.0
mmu-miR-378d	6.76E-03	-1.6	TargetScan	Moderate (predicted)	1456003_a_at	SLC1A4	1.00E-04	2.9
mmu-miR-378d	6.76E-03	-1.6	TargetScan	Moderate (predicted)	1426663_s_at	SLC45A3	5.00E-04	3.7
mmu-miR-378d	6.76E-03	-1.6	TargetScan	Moderate (predicted)	1420372_at	SNTB2	2.80E-03	2.0
mmu-miR-362-3p	2.30E-02	-1.7	TargetScan	Moderate (predicted)	1438431_at	ABCD2	1.00E-04	6.3
mmu-miR-362-3p	2.30E-02	-1.7	TargetScan	Moderate (predicted)	1417066_at	ADCK3	1.00E-03	2.1
mmu-miR-362-3p	2.30E-02	-1.7	TargetScan	Moderate (predicted)	1450883_a_at	CD36	0.00E00	4.4
mmu-miR-362-3p	2.30E-02	-1.7	TargetScan	Moderate (predicted)	1437313_x_at	HMGB2	4.60E-03	3.4
mmu-miR-362-3p	2.30E-02	-1.7	TargetScan	High (predicted)	1439038_at	KHNYN	4.62E-02	2.3
mmu-miR-362-3p	2.30E-02	-1.7	TargetScan	High (predicted)	1444487_at	LRAT	3.66E-02	2.1
mmu-miR-362-3p	2.30E-02	-1.7	TargetScan	Moderate (predicted)	1434853_x_at	MKRN1	1.00E-04	2.5
mmu-miR-362-3p	2.30E-02	-1.7	TargetScan	Moderate (predicted)	1434628_a_at	RHPN2	0.00E00	3.4
mmu-miR-362-3p	2.30E-02	-1.7	TargetScan	Moderate (predicted)	1441624_at	SORBS2	1.00E-03	2.0
mmu-miR-362-3p	2.30E-02	-1.7	TargetScan	High (predicted)	1448666_s_at	TOB2	1.00E-04	3.5
mmu-miR-3473b	2.35E-02	3.7	TargetScan	High (predicted)	1453023_at	ANKHD1/ANKHD1-EIF4EBP3	1.49E-02	-2.6
mmu-miR-3473b	2.35E-02	3.7	TargetScan	Moderate (predicted)	1451615_at	CES4A	1.40E-03	-3.3
mmu-miR-3473b	2.35E-02	3.7	TargetScan	Moderate (predicted)	1417991_at	DIO1	1.78E-02	-6.0
mmu-miR-3473b	2.35E-02	3.7	TargetScan	Moderate (predicted)	1437554_at	PLEC	2.57E-02	-2.2
mmu-miR-3473b	2.35E-02	3.7	TargetScan	Moderate (predicted)	1457794_at	WHSC1L1	2.62E-02	-2.0
mmu-miR-361-5p	2.25E-02	-1.6	TargetScan	High (predicted)	1454918_at	AGPS	0.00E00	2.2
mmu-miR-361-5p	2.25E-02	-1.6	TargetScan	Moderate (predicted)	1454617_at	ARRDC3	2.42E-02	2.3
mmu-miR-361-5p	2.25E-02	-1.6	TargetScan	High (predicted)	1439012_a_at	DCK	4.19E-02	2.2
mmu-miR-361-5p	2.25E-02	-1.6	TargetScan	High (predicted)	1427938_at	MYCBP	1.00E-04	2.3
mmu-miR-361-5p	2.25E-02	-1.6	TargetScan	High (predicted)	1417273_at	PDK4	3.50E-03	2.5
mmu-miR-361-5p	2.25E-02	-1.6	TargetScan	High (predicted)	1418760_at	RDH11	1.60E-03	3.2
mmu-miR-361-5p	2.25E-02	-1.6	TargetScan	High (predicted)	1434520_at	SC5D	8.90E-03	2.6

mmu-miR-361-5p	2.25E-02	-1.6	TargetScan	High (predicted)	1420372_at	SNTB2	2.80E-03	2.0
mmu-miR-361-5p	2.25E-02	-1.6	TargetScan	Moderate (predicted)	1420447_at	SULT1E1	2.50E-03	53.0
mmu-miR-365-3p	4.31E-03	-2.1	TargetScan	High (predicted)	1453289_at	AGO4	9.60E-03	2.0
mmu-miR-365-3p	4.31E-03	-2.1	TargetScan	High (predicted)	1436967_at	ANKRD11	3.97E-02	3.1
mmu-miR-365-3p	4.31E-03	-2.1	TargetScan	High (predicted)	1454617_at	ARRDC3	2.42E-02	2.3
mmu-miR-365-3p	4.31E-03	-2.1	TargetScan	Moderate (predicted)	1460196_at	CBR1	1.90E-03	2.2
mmu-miR-365-3p	4.31E-03	-2.1	TargetScan	High (predicted)	1417420_at	CCND1	6.00E-04	2.7
mmu-miR-365-3p	4.31E-03	-2.1	TargetScan	Moderate (predicted)	1424638_at	CDKN1A	1.09E-02	6.9
mmu-miR-365-3p	4.31E-03	-2.1	TargetScan	High (predicted)	1422578_at	CS	1.00E-04	2.0
mmu-miR-365-3p	4.31E-03	-2.1	TargetScan	Moderate (predicted)	1418989_at	CTSE	0.00E00	2.1
mmu-miR-365-3p	4.31E-03	-2.1	TargetScan	Moderate (predicted)	1438169_a_at	FRMD4B	2.00E-04	3.6
mmu-miR-365-3p	4.31E-03	-2.1	TargetScan	Moderate (predicted)	1427229_at	HMGCR	4.80E-03	4.1
mmu-miR-365-3p	4.31E-03	-2.1	TargetScan	High (predicted)	1433446_at	HMGCS1	9.00E-04	2.7
mmu-miR-365-3p	4.31E-03	-2.1	TargetScan	High (predicted)	1435319_at	IP6K2	7.00E-04	2.4
mmu-miR-365-3p	4.31E-03	-2.1	TargetScan	Moderate (predicted)	1425673_at	LPP	5.00E-04	2.1
mmu-miR-365-3p	4.31E-03	-2.1	TargetScan	High (predicted)	1427938_at	MYCBP	1.00E-04	2.3
mmu-miR-365-3p	4.31E-03	-2.1	TargetScan	Moderate (predicted)	1419820_at	PKHD1	2.90E-03	2.5
mmu-miR-365-3p	4.31E-03	-2.1	TargetScan	Moderate (predicted)	1417963_at	PLTP	0.00E00	4.5
mmu-miR-365-3p	4.31E-03	-2.1	TargetScan	Moderate (predicted)	1453578_at	PTER	6.00E-04	2.2
mmu-miR-365-3p	4.31E-03	-2.1	TargetScan	High (predicted)	1448754_at	RBP1	4.93E-02	2.4
mmu-miR-365-3p	4.31E-03	-2.1	TargetScan	Moderate (predicted)	1418760_at	RDH11	1.60E-03	3.2
mmu-miR-365-3p	4.31E-03	-2.1	TargetScan	High (predicted)	1437983_at	SALL1	4.10E-03	2.6
mmu-miR-365-3p	4.31E-03	-2.1	TargetScan	Moderate (predicted)	1433473_x_at	TM2D2	0.00E00	2.3
mmu-miR-466i-3p	2.47E-03	3.5	TargetScan	Moderate (predicted)	1436229_at	FAM126B	5.00E-03	-2.1
mmu-miR-466i-3p	2.47E-03	3.5	TargetScan	Moderate (predicted)	1450063_at	FMN2	9.00E-04	-3.7
mmu-miR-466i-3p	2.47E-03	3.5	TargetScan	Moderate (predicted)	1428820_at	MAPRE1	1.00E-04	-4.0
mmu-miR-466i-3p	2.47E-03	3.5	TargetScan	Moderate (predicted)	1436736_x_at	NREP	9.60E-03	-5.8
mmu-miR-466i-3p	2.47E-03	3.5	TargetScan	Moderate (predicted)	1418835_at	PHLDA1	1.00E-04	-5.8

mmu-miR-466i-3p	2.47E-03	3.5	TargetScan	Moderate (predicted)	1457695_at	SYNRG	4.04E-02	-2.1
mmu-miR-466i-3p	2.47E-03	3.5	TargetScan	High (predicted)	1456094_at	USP36	2.59E-02	-3.3
mmu-miR-423-5p	1.57E-02	-1.6	TargetScan	High (predicted)	1434185_at	ACACA	8.50E-03	2.5
mmu-miR-423-5p	1.57E-02	-1.6	TargetScan	Moderate (predicted)	1422925_s_at	ACOT1	3.50E-03	5.7
mmu-miR-423-5p	1.57E-02	-1.6	TargetScan	Moderate (predicted)	1439478_at	ACOT2	2.60E-03	2.2
mmu-miR-423-5p	1.57E-02	-1.6	TargetScan	Moderate (predicted)	1418250_at	ARL4D	2.40E-03	2.6
mmu-miR-423-5p	1.57E-02	-1.6	TargetScan	Moderate (predicted)	1439109_at	CCDC68	3.60E-03	2.2
mmu-miR-423-5p	1.57E-02	-1.6	TargetScan	Moderate (predicted)	1418126_at	CCL5	1.30E-02	6.4
mmu-miR-423-5p	1.57E-02	-1.6	TargetScan	High (predicted)	1424638_at	CDKN1A	1.09E-02	6.9
mmu-miR-423-5p	1.57E-02	-1.6	TargetScan	Moderate (predicted)	1435207_at	DIXDC1	9.00E-04	2.2
mmu-miR-423-5p	1.57E-02	-1.6	TargetScan	High (predicted)	1420965_a_at	ENC1	0.00E00	3.1
mmu-miR-423-5p	1.57E-02	-1.6	TargetScan	Moderate (predicted)	1441881_x_at	FAM101A/ZNF664-FAM101A	1.40E-03	3.2
mmu-miR-423-5p	1.57E-02	-1.6	TargetScan	Moderate (predicted)	1448354_at	G6PD	6.00E-04	2.4
mmu-miR-423-5p	1.57E-02	-1.6	TargetScan	High (predicted)	1439264_x_at	LASP1	2.00E-04	5.8
mmu-miR-423-5p	1.57E-02	-1.6	TargetScan	Moderate (predicted)	1439269_x_at	MCM7	2.81E-02	2.1
mmu-miR-423-5p	1.57E-02	-1.6	TargetScan	High (predicted)	1454903_at	NGFR	2.60E-03	2.1
mmu-miR-423-5p	1.57E-02	-1.6	TargetScan	High (predicted)	1417689_a_at	PDZK1IP1	7.00E-04	2.9
mmu-miR-423-5p	1.57E-02	-1.6	TargetScan	Moderate (predicted)	1417963_at	PLTP	0.00E00	4.5
mmu-miR-423-5p	1.57E-02	-1.6	TargetScan	Moderate (predicted)	1421382_at	PRLR	1.00E-04	2.9
mmu-miR-423-5p	1.57E-02	-1.6	TargetScan	Moderate (predicted)	1421430_at	RAD51B	1.01E-02	11.7
mmu-miR-423-5p	1.57E-02	-1.6	TargetScan	Moderate (predicted)	1438617_at	SERPINA7	1.90E-03	3.3
mmu-miR-423-5p	1.57E-02	-1.6	TargetScan	High (predicted)	1428338_at	SPATA2L	5.10E-03	2.1
mmu-miR-423-5p	1.57E-02	-1.6	TargetScan	Moderate (predicted)	1450918_s_at	SRC	5.70E-03	2.0
mmu-miR-423-5p	1.57E-02	-1.6	TargetScan	Moderate (predicted)	1438855_x_at	TNFAIP2	2.00E-04	2.8
mmu-miR-425-5p	1.83E-02	-1.9	TargetScan	Moderate (predicted)	1426392_a_at	ACTR3	0.00E00	2.3
mmu-miR-425-5p	1.83E-02	-1.9	TargetScan	High (predicted)	1447606_x_at	AQP11	0.00E00	2.7
mmu-miR-425-5p	1.83E-02	-1.9	TargetScan	Moderate (predicted)	1428512_at	BHLHB9	2.43E-02	2.2
mmu-miR-425-5p	1.83E-02	-1.9	TargetScan	High (predicted)	1417420_at	CCND1	6.00E-04	2.7

mmu-miR-425-5p	1.83E-02	-1.9	TargetScan	Moderate (predicted)	1450883_a_at	CD36	0.00E00	4.4
mmu-miR-425-5p	1.83E-02	-1.9	TargetScan	Moderate (predicted)	1430997_at	CD47	1.30E-03	2.5
mmu-miR-425-5p	1.83E-02	-1.9	TargetScan	High (predicted)	1434272_at	CPEB2	1.80E-03	2.0
mmu-miR-425-5p	1.83E-02	-1.9	TargetScan	High (predicted)	1421365_at	FST	2.19E-02	3.6
mmu-miR-425-5p	1.83E-02	-1.9	TargetScan	Moderate (predicted)	1455417_at	KCNJ11	4.95E-02	3.2
mmu-miR-425-5p	1.83E-02	-1.9	TargetScan	High (predicted)	1425673_at	LPP	5.00E-04	2.1
mmu-miR-425-5p	1.83E-02	-1.9	TargetScan	Moderate (predicted)	1428167_a_at	MPZL1	1.48E-02	2.1
mmu-miR-425-5p	1.83E-02	-1.9	TargetScan	Moderate (predicted)	1431332_a_at	TERF1	1.50E-03	2.1
mmu-miR-451a	1.07E-02	-1.9	miRecords	Experimentally Observed	1419758_at	ABCB1	1.70E-03	3.5
mmu-miR-451a	1.07E-02	-1.9	TargetScan	Moderate (predicted)	1456590_x_at	AKR1B1	0.00E00	2.5
mmu-miR-451a	1.07E-02	-1.9	TargetScan	Moderate (predicted)	1424250_a_at	ARHGEF3	2.00E-04	2.4
mmu-miR-451a	1.07E-02	-1.9	TargetScan	Moderate (predicted)	1418918_at	IGFBP1	8.30E-03	12.4
mmu-miR-451a	1.07E-02	-1.9	TargetScan	Moderate (predicted)	1426913_at	LSS	3.60E-03	2.7
mmu-miR-467f	6.64E-06	3.1	TargetScan	High (predicted)	1418496_at	FOXA1	2.50E-03	-2.8
mmu-miR-467f	6.64E-06	3.1	TargetScan	Moderate (predicted)	1453596_at	ID2	2.20E-03	-2.5
mmu-miR-467f	6.64E-06	3.1	TargetScan	Moderate (predicted)	1433408_a_at	MCM10	4.00E-04	-3.2
mmu-miR-467f	6.64E-06	3.1	TargetScan	High (predicted)	1438597_x_at	MORF4L1	3.30E-03	-2.1
mmu-miR-467f	6.64E-06	3.1	TargetScan	Moderate (predicted)	1436366_at	PPP1R15B	2.82E-02	-2.0
mmu-miR-467f	6.64E-06	3.1	TargetScan	Moderate (predicted)	1447700_x_at	SS18L1	0.00E00	-10.1
mmu-miR-467f	6.64E-06	3.1	TargetScan	Moderate (predicted)	1456094_at	USP36	2.59E-02	-3.3
mmu-miR-3107-5p	2.52E-04	-2.5	TargetScan	High (predicted)	1447606_x_at	AQP11	0.00E00	2.7
mmu-miR-3107-5p	2.52E-04	-2.5	TargetScan	Moderate (predicted)	1459937_at	ASS1	9.40E-03	2.0
mmu-miR-3107-5p	2.52E-04	-2.5	TargetScan	High (predicted)	1422439_a_at	CDK4	1.50E-03	2.0
mmu-miR-3107-5p	2.52E-04	-2.5	TargetScan	High (predicted)	1428372_at	ST5	3.00E-04	2.3
mmu-miR-7a-5p	1.54E-02	-1.9	TargetScan	High (predicted)	1454918_at	AGPS	0.00E00	2.2
mmu-miR-7a-5p	1.54E-02	-1.9	TargetScan	High (predicted)	1434272_at	CPEB2	1.80E-03	2.0
mmu-miR-7a-5p	1.54E-02	-1.9	TargetScan	High (predicted)	1428306_at	DDIT4	3.20E-03	2.5
mmu-miR-7a-5p	1.54E-02	-1.9	TargetScan	Moderate (predicted)	1438169_a_at	FRMD4B	2.00E-04	3.6

mmu-miR-7a-5p	1.54E-02	-1.9	TargetScan	High (predicted)	1439424_x_at	HERPUD2	2.20E-03	4.4
mmu-miR-7a-5p	1.54E-02	-1.9	TargetScan	High (predicted)	1425673_at	LPP	5.00E-04	2.1
mmu-miR-7a-5p	1.54E-02	-1.9	TargetScan	Moderate (predicted)	1416750_at	SIGMAR1	6.00E-04	2.0
mmu-miR-7a-5p	1.54E-02	-1.9	TargetScan	High (predicted)	1448502_at	SLC16A7	2.90E-03	2.0
mmu-miR-7a-5p	1.54E-02	-1.9	TargetScan	Moderate (predicted)	1433473_x_at	TM2D2	0.00E00	2.3
mmu-miR-25-3p	7.47E-03	-1.9	TargetScan	High (predicted)	1454617_at	ARRDC3	2.42E-02	2.3
mmu-miR-25-3p	7.47E-03	-1.9	TargetScan	High (predicted)	1451306_at	CDCA7L	2.14E-02	2.4
mmu-miR-25-3p	7.47E-03	-1.9	Ingenuity Expert Findings	Experimentally Observed	1424638_at	CDKN1A	1.09E-02	6.9
mmu-miR-25-3p	7.47E-03	-1.9	TargetScan	High (predicted)	1434272_at	CPEB2	1.80E-03	2.0
mmu-miR-25-3p	7.47E-03	-1.9	TargetScan	High (predicted)	1428306_at	DDIT4	3.20E-03	2.5
mmu-miR-25-3p	7.47E-03	-1.9	TargetScan	High (predicted)	1439424_x_at	HERPUD2	2.20E-03	4.4
mmu-miR-25-3p	7.47E-03	-1.9	TargetScan	High (predicted)	1454671_at	INSIG1	4.00E-04	2.2
mmu-miR-25-3p	7.47E-03	-1.9	TargetScan	High (predicted)	1425673_at	LPP	5.00E-04	2.1
mmu-miR-25-3p	7.47E-03	-1.9	TargetScan	High (predicted)	1437527_x_at	MCL1	3.80E-03	2.3
mmu-miR-25-3p	7.47E-03	-1.9	TargetScan	Moderate (predicted)	1425434_a_at	MSR1	4.62E-02	2.0
mmu-miR-25-3p	7.47E-03	-1.9	TargetScan	Moderate (predicted)	1420984_at	PCTP	7.00E-04	5.5
mmu-miR-25-3p	7.47E-03	-1.9	TargetScan	Moderate (predicted)	1421430_at	RAD51B	1.01E-02	11.7
mmu-miR-25-3p	7.47E-03	-1.9	TargetScan	High (predicted)	1434628_a_at	RHPN2	0.00E00	3.4
mmu-miR-25-3p	7.47E-03	-1.9	TargetScan	High (predicted)	1426663_s_at	SLC45A3	5.00E-04	3.7
mmu-miR-25-3p	7.47E-03	-1.9	TargetScan	Moderate (predicted)	1433473_x_at	TM2D2	0.00E00	2.3
mmu-miR-25-3p	7.47E-03	-1.9	TargetScan	High (predicted)	1448666_s_at	TOB2	1.00E-04	3.5
mmu-miR-25-3p	7.47E-03	-1.9	TargetScan	High (predicted)	1424669_at	ZFYVE21	1.00E-04	2.1

Supplementary Table S10. mRNA-specific primers used for qRT-PCR.

Gene symbol	Forward primer (5' –> 3')	Reverse primer (5' –> 3')
Fmo3	AAGCCTATGAAGACGCGTG	GGACTTATGCCCACTGCAGAT
Apoa4	CCAATGTGGTGTGGGATTACTT	AGTGACATCCGTCTTCTGAAAC
Hspa1b	GACGAGGGTCTCAAGGGCA	TTCTAGACCACACCGGGAG
Ugt2b38	ATTCACCACGACCAGCCTA	TGAACAAGAGGCACTTTACAG
Elovl3	ACATCTGGAGGCAGGAGAA	CCATACAGGGAGGTACCCT
Gapdh	ACCAGGTTGTCTCCTGCGA	CAGTGTCCTTGCTGGGGTG

Supplementary Table S11. miRNA-specific primers used for qRT-PCR.

miRNA	Primer (5'–> 3')
miR-1927	GACCTCTGGATGTTAGGGACTGA
miR-802-5p	TCAGTAACAAAGATTCATCCTT
miR-6236	GCCGTCGCCGGCAGTCA
miR-3968	CGAATCCCACTCCAGACACCA
miR-126a-5p	CATTATTACTTTTGGTACGCG
miR-100-5p	AACCCGTAGATCCGAACTTG
miR-5100	TCGAATCCCAGCGGTGCCT
miR-3102-5p	GTGAGTGGCCAGGGTGGGGCTG
U6	CAAGGATGACACGCAAATTCG
